# Epigenetically regulated digital signaling defines epithelial innate immunity at the tissue level

**DOI:** 10.1038/s41467-021-22070-x

**Published:** 2021-03-23

**Authors:** Helen R. Clark, Connor McKenney, Nathan M. Livingston, Ariel Gershman, Seema Sajjan, Isaac S. Chan, Andrew J. Ewald, Winston Timp, Bin Wu, Abhyudai Singh, Sergi Regot

**Affiliations:** 1 Department of Molecular Biology and Genetics, Johns Hopkins University School of Medicine, Baltimore, MD USA; 2 Oncology Department, Johns Hopkins University School of Medicine, Baltimore, MD USA; 3 The Biochemistry, Cellular, and Molecular Biology Graduate Program, Johns Hopkins University School of Medicine, Baltimore, MD USA; 4 Center for Cell Dynamics, Johns Hopkins University School of Medicine, Baltimore, MD USA; 5 Department of Biophysics and Biophysical Chemistry, Johns Hopkins School of Medicine, Baltimore, MD USA; 6 Department of Biomedical Engineering, Johns Hopkins University, Baltimore, MD USA; 7 Department of Cell Biology, Johns Hopkins University School of Medicine, Baltimore, MD USA; 8 Electrical and Computer Engineering, University of Delaware, Newark, DE USA

**Keywords:** Cell signalling, Epigenetics, Innate immunity, Pattern recognition receptors

## Abstract

To prevent damage to the host or its commensal microbiota, epithelial tissues must match the intensity of the immune response to the severity of a biological threat. Toll-like receptors allow epithelial cells to identify microbe associated molecular patterns. However, the mechanisms that mitigate biological noise in single cells to ensure quantitatively appropriate responses remain unclear. Here we address this question using single cell and single molecule approaches in mammary epithelial cells and primary organoids. We find that epithelial tissues respond to bacterial microbe associated molecular patterns by activating a subset of cells in an all-or-nothing (i.e. digital) manner. The maximum fraction of responsive cells is regulated by a bimodal epigenetic switch that licenses the TLR2 promoter for transcription across multiple generations. This mechanism confers a flexible memory of inflammatory events as well as unique spatio-temporal control of epithelial tissue-level immune responses. We propose that epigenetic licensing in individual cells allows for long-term, quantitative fine-tuning of population-level responses.

## Abstract (web summary)

Fine tuning the immune response in line with the degree of threat is central to recognition of a pathogen versus commensal bacteria. Here the authors implicate a digital all-or-nothing cell response in control of the tissue level immune response.

## Introduction

At the cellular juncture of host and environment, epithelial tissues interact with a wide variety of microorganisms via pattern recognition receptors (i.e. Toll-Like Receptors)^1^. Precise regulation of Toll-Like Receptor (TLR) signaling is critical—impaired responses invite persistent infection while excessive responses trigger autoimmunity, septic shock, or chronic inflammation^2^. To strike a balance, epithelial tissues regulate innate immune signaling in multiple ways: (i) receptor expression in intestinal epithelia is restricted to specific regions, cell types, or subcellular compartments^3,4^; (ii) cytokines produced during chronic inflammation (e.g. irritable bowel syndrome, or cystic fibrosis)^5,6^ upregulate TLR expression and; (iii) epithelial cells become tolerant to repeated TLR stimulation^7^. These multiple layers of regulation highlight the dynamic nature of the epithelial innate immune system at both the tissue and single-cell levels, yet how these mechanisms are temporally coordinated to ensure quantitatively appropriate responses remains poorly understood.

TLR signaling in epithelial cells results in nuclear translocation of the transcription factor NF-κB (Nuclear Factor Kappa B), which initiates and drives the gene expression program necessary to mount an inflammatory response^8^. Live tracking of NF-κB nuclear translocation in response to different ligands has revealed a surprising degree of single-cell heterogeneity in dynamic patterns of activity. Certain stimuli produce continuous nucleocytoplasmic shuttling, while others trigger a single transient pulse^9–14^. These temporal behaviors result from the combined action of specific signaling intermediates such as MyD88, IRAK1, TRADD, or TRIF, and transcriptionally induced negative regulators such as IκBα or A20^15–18^. NF-κB dynamics have been shown to specify gene expression patterns^19,20^ and mitigate cell-to-cell variation^21^ in different systems but the role of continued vs. transient NF-κB activation in the context of epithelial tissues remains unclear.

At the tissue level, previous studies have shown that innate immune responses are modulated through epigenetic reprogramming^22^. Chromatin and DNA modifications specify gene expression programs over multiple generations, providing memory and tolerance to the immune system^23^. Recent studies of allele-specific methylation indicate that genomic loci stochastically switch between two metastable states –completely methylated, or completely unmethylated^24^. These bistable switches play an important role in cancer and drug resistance through the generation of phenotypic heterogeneity^25,26^. Epigenetic switching also plays a critical role in T-cell fate specification^27^. However, the role of epigenetic switches in shaping innate immune responses at the single-cell level has not been addressed.

To better understand how epithelial tissues quantitatively evaluate stimuli we measured NF-κB signaling and gene expression dynamics in single cells within epithelial monolayers. We found that in both, cell lines and primary organoids, bacterial lipopeptides activate only a subset of cells. While stimuli concentration tunes the fraction of responding cells^28^, the maximum proportion of responsive cells is dictated by a bistable epigenetic switch controlling bimodal TLR2 expression. Interestingly, the switching probabilities are regulated by cytokines that trigger sustained NF-κB oscillations (i.e. TNFα), creating a positive feedback loop at the tissue level. Thus, bistable epigenetic control of signaling components in individual cells allows for quantitative regulation of the innate immune response at the tissue level.

## Results

### Single-cell analysis of NF-κB dynamics reveals digital signaling within epithelial monolayers

**Fig. 1:**
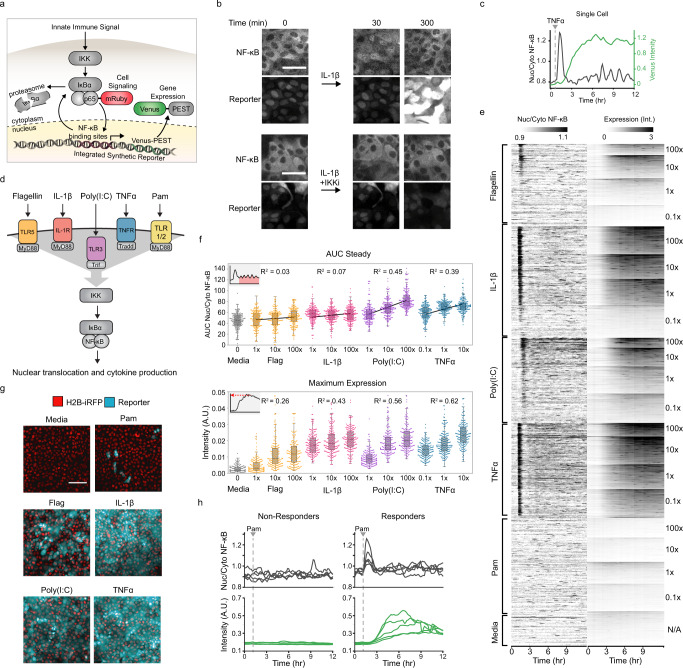
MAMP sensing in epithelial monolayers shows digital and analog components. **a** Schematic detailing the NF-κB-dependent gene expression reporter system. An innate immune signal activates IKK which phosphorylates IκBα and sends it for degradation, freeing the fluorescently tagged NF-κB subunit (p65-mRuby) to enter the nucleus and transcribe the Venus-PEST reporter. **b** MCF10A NF-κB reporter cells were incubated in the presence or absence of IL-1β (10 ng/ml) and/or IKK inhibitor VII (10 µM). Representative images from five replicates taken at indicated times are shown. Scale bar, 50 μm. **c** Representative single-cell trace. The median intensity of the nuclear/cytoplasmic NF-κB is plotted in black, while the median nuclear + cytoplasmic intensity gene expression reporter is in green. **d** Schematic of innate immune receptors and inputs that signal to NF-κB. TLR5, IL1R, and TLR1/2 all signal through MyD88. **e** Heatmap of all cells in the MAMP screen. MCF10A monoclonal reporter cell line was imaged for 50 min then treated with the indicated input and concentration. Concentrations at 100x were 1 µg/ml (Flagellin), 200 µg/ml (Poly(I:C)), 1 µg/ml (Pam3CSK4), 1 µg/ml (TNFα), and 100 ng/ml (IL-1β). Heatmaps are ordered top to bottom from highest to lowest maximum Venus expression. **f** (Top) Swarmplots of the area under the curve after the first NF-κB translocation peak (AUC steady) in 200 cells randomly sampled from the indicated input and concentration. (Bottom) Swarmplots of the 95th percentile of the gene expression in 200 cells randomly sampled from the indicated input and concentration. R squared values of the linear regression model fit to responses across the log of three concentrations. **g** Representative images of clonal MCF10A after 8 h of incubation with indicated stimulus (1 µg/ml (Flagellin), 100 ng/ml (IL-1β), 20 µg/ml (Poly(I:C)), 100 ng/ml (TNFα), and 1 µg/ml (Pam3CSK4)). H2B-iRFP nuclear marker is shown in red and Venus gene expression reporter is shown in cyan. Scale bar, 200 µm. **h** MCF10A NF-κB reporter cells stimulated with 1 µg/ml Pam3CSK4. Traces of NF-κB translocation (gray) and Venus expression (green) for five non-responders and responders are shown.

To study how epithelial tissues quantify innate immune signals we generated an epithelial cell line that allows simultaneous measurements of NF-κB localization and target gene expression in real-time and with single-cell resolution. We isolated a clone of the chromosomally normal, human mammary epithelial cell line, MCF10A, expressing a fluorescently tagged NF-κB (p65-mRuby), a nuclear marker (H2B-iRFP) and Venus-PEST^29^ under the control of a synthetic NF-κB promoter (7x NF-κB binding sites; Fig. 1a). To validate the reporter system we treated cells with IL-1β in the presence or absence of an inhibitor of the upstream IκBα kinase (IKK). As expected, IL-1β addition led to NF-κB nuclear translocation as well as Venus reporter expression. Both processes were effectively blocked by the IKK inhibitor (Fig. 1b). Use of this reporter cell line and custom image analysis software allowed the simultaneous quantification of NF-κB signaling (nuclear/cytoplasmic p65 intensity ratio) and gene expression (Venus intensity) in individual cells (Fig. 1c and Supplementary Movie 1). To better understand the correlations between stimuli concentration, signaling, and gene expression, we used live imaging of the reporter system to screen responses to a panel of stimuli that activate distinct branches of the NF-κB network: Flagellin (TLR5), IL-1β (IL1R), Poly(I:C)(TLR3), TNFα (TNFR), and Pam3CSK4 (TLRs 1/2; Fig. 1d). We treated cells with ligands at different concentrations spanning four orders of magnitude and collected data from more than 18,000 cells (Fig. 1e). Notably, cells treated with 10- or 100-fold different stimuli concentration showed a significant overlap in response amplitude, suggesting poor quantitative discrimination at the single-cell level (Fig. 1e).

To further quantify the correlation between signaling, and gene expression we extracted multiple parameters describing the response of each individual cell and conducted a comprehensive correlation analysis (Supplementary Fig. 1a). Results confirmed that signaling and gene expression are not well correlated at the single-cell level using any of these parameters (max *R* = 0.328). However, two distinct signaling patterns emerged. While flagellin (TLR5) and IL-1β (IL1R) rapidly quench nuclear NF-κB after the initial peak and show little to no steady-state activity, dsRNA (TLR3) and TNFα (TNFR) create dose-dependent oscillatory NF-κB activity that continues for hours (Fig. 1f). The stimuli that created sustained oscillatory NF-κB activity also showed a higher correlation of gene expression with input concentration (Fig. 1f). CRISPR knockouts of signaling intermediates TRADD, MyD88, and TRIFF confirmed that TLR5 and IL1R rely on MyD88 for signal transduction while TLR3 and TNFR depend on TRIFF and TRADD respectively (Supplementary Fig. 1b). Therefore, our results suggest that the ability to maintain high steady-state NF-κB signaling over time correlates with input concentration and signaling intermediates.

**Fig. 2:**
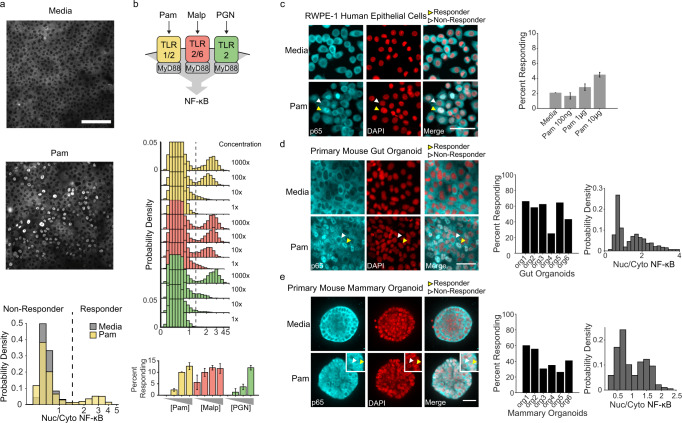
Pam3CSK4 triggers a capped digital response in primary epithelial cells and organoids. **a** p65 immunofluorescent stain of wild type MCF10A stimulated with media (top) or 1 µg/ml Pam3CSK4 (bottom) for 30 min. Scale bar, 300 µm. Histogram shows nuclear/cytoplasmic p65 intensity in logarithmic scale. Cells with nuclear/cytoplasmic p65 ratio greater than media controls were considered responders. **b** Dose-response curve of WT MCF10A with 30 minutes of Pam3CSK4 (0.01, 0.1, 1, 10 µg/ml), MALP (0.001, 0.01, 0.1, 1 µg/ml), or PGN (0.01, 0.1, 1, 10 µg/ml). Data represent the mean ± SD of three replicates. **c** Human prostate epithelial cells (RWPE-1) were cultured, treated for 30 mins with 10 µg/ml Pam3CSK4, fixed and stained as described in methods. Representative images are shown. Yellow and white arrows indicate examples of responder and non-responder cells respectively. Scale bar, 50 µm. Percent of cells in monolayer responding to increasing concentrations of Pam3CSK4. Data represent the mean ± SD of four replicates. **d** Primary mouse gut organoids were cultured as described in methods, treated for 30 mins with 10 µg/ml Pam3CSK4 and immunostained. Representative images are shown. Scale bar, 100 µm. Bar plot and histograms show quantification of percent responding cells in individual organoids and nuclear/cytoplasmic NF-κB amplitude of response in combined organoids. **e** Primary mouse mammary organoids were cultured as described in methods, treated for 30 mins with 10 µg/ml Pam3CSK4 and immunostained. Representative images of three independent replicates are shown. Scale bar, 30 µm. Bar plot and histograms show quantification as in panel **d**.

Surprisingly, while most stimuli led to uniform Venus expression across the monolayer, the TLR5 and the TLR1/2 agonists flagellin and bacterial lipopeptide Pam3CSK4 (Pam), triggered a response in only a subset of the cells (Fig. 1g, h and Supplementary Movie 2). Further analysis of the signaling amplitude of the responders showed that TLR5 stimulation generated a continuum of weak amplitude responders at high concentrations, but TLR1/2 stimulation showed bimodal (i.e. all-or-nothing) behavior (Fig. 2a and Supplementary Fig. 2a). Taken together our data suggest that while individual cells show poor quantitative discrimination of stimuli concentration, at the tissue level, activation of different groups of cells may enable more quantitative innate immune responses (Supplementary Fig. 2b). Thus, we decided to investigate the mechanism underlying digital NF-κB signaling as well as its potential regulation.

### Digital NF-κB activation occurs in isogenic clones and primary organoids

To study the differences between responders and non-responders in epithelial monolayers we first asked if genetic heterogeneity underlies digital NF-κB signaling. To address this question, we generated multiple subclones of our reporter line and determined the fraction of responders. Even in isogenic clones, the fraction of Pam-responding cells was similar to the parental cell line (Supplementary Fig. 3a). Immunofluorescent staining for NF-κB revealed that even in WT MCF10A cells without any reporter the response is highly bimodal with ~15% responders (Fig. 2a). This number was significantly higher than our reporter cell line, suggesting that lentiviral transduction may impact the steady-state fraction of responders.

In addition to mediating the Pam response, TLR2 is capable of forming heterodimers with other TLRs to detect a diverse spectrum of Microbe Associated Molecular Patterns (MAMPs) from bacteria, fungi, viruses, and host danger signals^30^. Therefore, we asked if other TLR2 agonists generate bimodal responses and found that both MALP and peptidoglycan also trigger digital NF-κB signaling. In all cases the fraction of responders reached a maximum of ~15% (Fig. 2b). To determine whether cells responding to different MAMPs represent the same or different subpopulations, we compared the percent of cells responding to singular and combined inputs and found that the fraction was similar, suggesting near complete overlap of these populations (Supplementary Fig. 3b).

Next we asked whether the digital response observed in MCF10A cells also occurs in other cell lines or primary organoids. We tested human cell lines derived from the three embryonic origins: ectodermal, mesenchymal, and endothelial. We found that while mesenchymal and endothelial cell types (BJs and HUVECs respectively) do not show any apparent response (Supplementary Fig. 4a), other epithelial cell types such as the prostate cell line RWPE-1 showed robust digital signaling in response to Pam (Fig. 2c). Additionally, we wanted to determine if digital NF-κB signaling also occurs in epithelial organoids derived from mouse. We cultured gut enteroids^31^ and mammary organoids^32^, and analyzed digital signaling by immunofluorescence. Interestingly, both epithelial organoids showed digital signaling at different levels (Fig. 2d, e and Supplementary Fig. 4b, c). These results suggest that NF-κB digital signaling occurs in multiple epithelial tissues in mammals.

### Responder status is epigenetically maintained

**Fig. 3:**
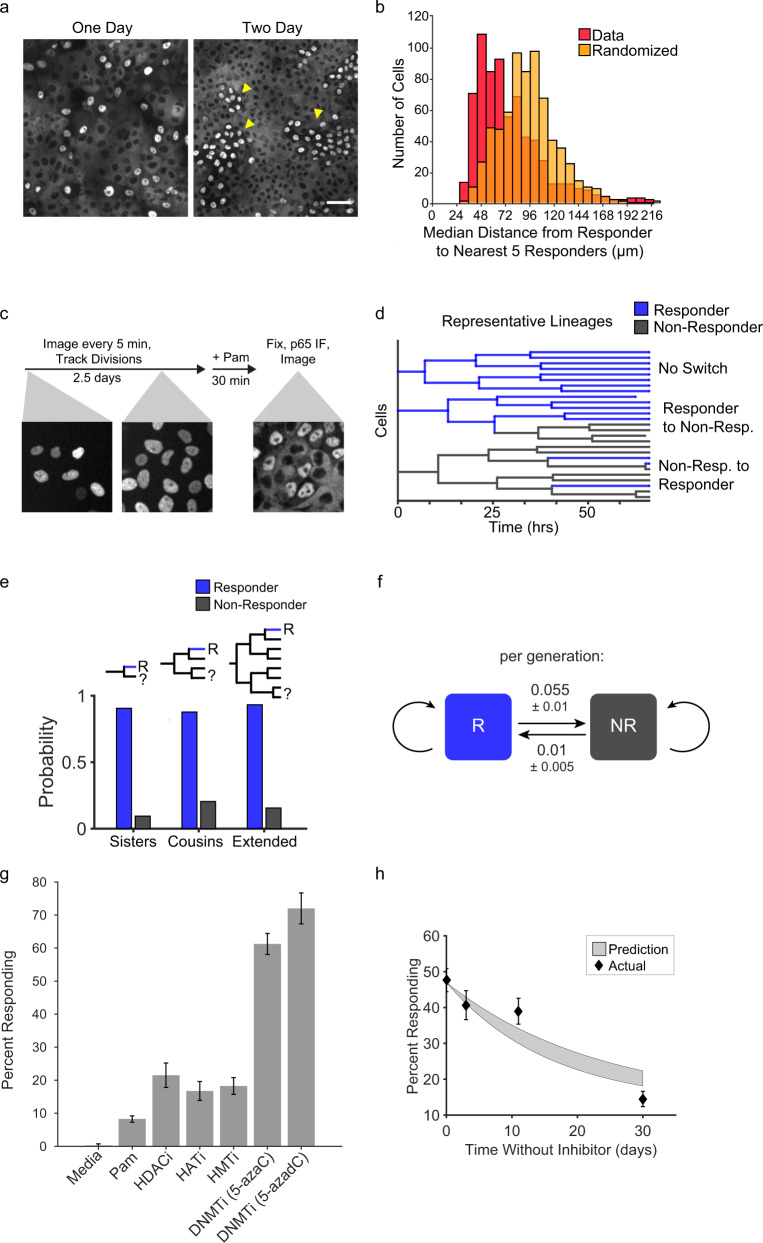
Responder cell status is maintained over multiple generations via epigenetic mechanisms. **a** WT MCF10A monolayers were grown for 24 (left) or 48 h (right), treated with 1 µg/ml Pam3CSK4, and immunostained for NF-κB. Arrows indicate clusters of responding cells. Scale bar, 100 µm. Images are representative of four replicates. **b** Monolayers were treated with 1 µg/ml Pam3CSK4 and immunostained for NF-κB response. Histograms show quantification of the median distance from each responder cell to the closest five responders in the monolayer versus randomized responder positions. **c** Schematic describing the workflow of the lineage-tracing experiment. Cell lineages were monitored for 60 h by tracking H2B-iRFP nuclear fluorescence. After the last time point cells were treated with 1 µg/ml Pam3CSK4, fixed and immunostained as described in Methods section. Responder status was measured in each terminal cell and the minimum number of status change events was assumed to reconstruct the lineage responder status. **d** Representative lineages obtained as described in panel **c** (Supplementary Fig. 5a). **e** Using data from lineage tracing the number of a responder cells having a sister, cousin, or extended relative cell that is a responder (blue) or non-responder (gray) was counted and the probability is reported. **f** Schematic to describe bistable switching rates obtained by model constructed from lineage tracing data. Responder and non-responder status is maintained with a low probability of switch per generation. See supplementary material for more information on the model. **g** WT MCF10A were cultured with epigenetic modifier inhibitors (HDACi (SAHA, 800 nM), HATi (A-485, 10 µM), HMTi (EPZ-6438, 5 µM), DNMTi (5-AzacytidineC, 500 nM, or 5-aza-2′-deoxycytidine (1 µM))) for 7 days prior to Pam3CSK4 treatment. Data represent the mean ± SD from four replicates. **h** Monolayers treated with 5-AzacytidineC as in panel g and cultured for 30 days from removal of the drug. At indicated times, the fraction of responder cells was measured as in Fig. 2a. Gray shading indicates the 95% confidence interval of the model prediction. Data represent the mean ± SD from three replicates.

In our attempts to determine the preexisting differences between responder and non-responder cells, we noted a positional bias for responders within the monolayer. The responder cells in a monolayer fixed after 1 day of growth were sparsely distributed, while multiple days of growth led to the formation of groups of responders in the monolayer (Fig. 3a). Accordingly, the median distance of a given responder to its responding neighbors was significantly lower when compared to a randomized control (Fig. 3b). This observation led us to speculate that the responder phenotype could be inherited within cell lineages and therefore more likely to be spatially segregated. We tested this hypothesis by live imaging and tracking cell lineages for three days prior to determining responder status by immunofluorescence (Fig. 3c). We tracked 273 divisions in 58 lineages that spanned up to five generations (Supplementary Fig. 5a) and confirmed that responders were more likely to be related to one another (Fig. 3d, e). However, there was a low probability of switching between the responding and non-responding states (Fig. 3f). We hypothesized that the responder fraction could be controlled by epigenetic mechanisms. To test this, we treated epithelial monolayers with methyltransferase, histone deacetylase (HDAC), histone acetyltransferase (HAT), and histone methyltransferase (HMT) inhibitors and found that the methyltransferase inhibitors 5-Azacytidine and 5-aza-2′-deoxycytidine significantly increased the fraction of responders to up to 60–70% of responders (Fig. 3g). In addition, the nuclear/cytoplasmic NF-κB amplitude of responders was comparable between treated and untreated responder cells (Supplementary Fig. 5b). We constructed a model based on stochastic switching between responder and non-responder states coupled to lineage expansion, where the mother cell state is inherited by daughters. Systematic model fitting to the lineage data revealed switching probabilities of 0.01 ± 0.005 (non-responder to responder) and 0.055 ± 0.01 (responder to non-responder) per generation (± denotes 95% confidence intervals, Fig. 3f; Supplementary Notes). These inferred switching probabilities predict the fraction of responders upon removal of the DNMT inhibitor, and the experimental data recapitulated the predicted rate of decay (Fig. 3h). These results suggest that DNA methylation is involved in maintaining the responder status. Next we sought to identify the epigenetically regulated signaling component involved in the response to Pam and other MAMPs.

### Bimodal expression of TLR2 creates digital response

**Fig. 4:**
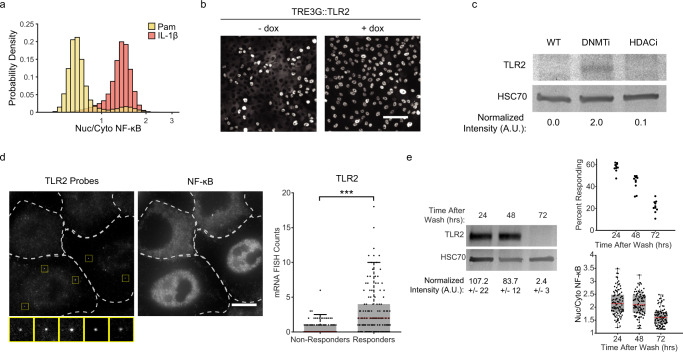
Bimodal TLR2 expression limits the fraction of responder cells. **a** Wild-type MCF10A were stimulated with 1 µg/ml Pam3CSK4 or 100 ng/ml IL-1β. Quantification of nuclear/cytoplasmic p65 immunofluorescent stain is shown in histograms on logarithmic scale. Data represents three replicates. **b** Cells were transduced with TLR2 under a Tet Responsive Element 3rd Generation (TRE3G) promoter and incubated with or without Doxycycline (2 µg/ml) for 24 h. Cells were then stimulated with 1 µg/ml Pam3CSK4 and immunostained to determine responder status as in Fig. 2a. Representative images of three replicates are shown. Scale bar, 100 µm. **c** Immunoblot against TLR2 in cells treated with or without DNMT inhibitor (5-AzacytidineC, 500 nM) or HDACi (SAHA 800 nM). HSC70 was used as a loading control. Data represents two replicates. **d** Dual smRNA FISH-immunofluorescence for TLR2 and NF-κB was done as described in Methods section. Dashed line indicates cell boundaries, yellow squares highlight the TLR2 probes. Representative images of two replicates are shown. Scale bar, 10 µm. Quantification of TLR2 mRNAs in responders and non-responders determined by NF-κB nuclear translocation. *N* = 354 cells total (148 Responder, 206 Non-Responder), *p* = 3.8e-19 by *χ*^2^ test with 9 degrees of freedom. **e** Western blot of TRE3G::TLR2 cells treated with a 4-h pulse of Doxycycline (2 µg/ml) and cultured for the indicated time. Relative amounts are normalized to HSC70 loading control. Standard deviation represents two independent experiments for each condition. Swarmplots represent technical replicates of the percentage of responding cells (top) and nucleus/cytoplasm NF-ĸB amplitude (bottom) in the same cells.

Previous reports have shown that the human TLR2 promoter is hypomethylated in disease states that involve excessive inflammation such as cystic fibrosis and periodontitis, increasing the expression level of the receptor^33,34^. Furthermore, our data show that TLR2 agonists trigger a MyD88-dependent digital response (Supplementary Fig. 6a), yet other MyD88-dependent innate immune signaling molecules such as IL-1β elicit a fully penetrant response (Fig. 4a). Therefore, we hypothesized that bimodal TLR2 expression could be involved in digital signaling. To address this question, we used a doxycycline-inducible promoter to drive heterologous expression of the TLR2 receptor and found that the percentage of pam-responding cells was increased to nearly 100% (Fig. 4b). We concluded that TLR2 expression limits the signaling capabilities of non-responding cells. If TLR2 expression is required in responder cells, population-level expression of the protein should be higher in scenarios where a larger portion of the monolayer is responsive, such as after treatment with 5-Azacytidine (Fig. 3g). Accordingly, immunoblotting for TLR2 showed that expression is significantly higher after treatment (Fig. 4c). These results suggested that TLR2 may be expressed in a subset of cells. To address this, we performed single-molecule mRNA fluorescence in situ hybridization (smRNA FISH) in tandem with NF-κB immunofluorescence in stimulated and unstimulated cells. Our results showed that responders contain significantly higher TLR2 mRNA counts when compared to non-responders (Fig. 4d). As a control, Pol II mRNA counts were indistinguishable between responder and non-responder cells (Supplementary Fig. 6b, c). Importantly, the distribution of TLR2 mRNA counts in stimulated and unstimulated cells was equivalent (Supplementary Fig. 6d). Together these data support the idea that within a monolayer, epigenetically regulated bimodal TLR2 expression limits the number of cells able to respond to bacterial lipopeptides.

While inhibition of epigenetic modifications affects the fraction of responders, we wondered if the stability of responder-status inheritance could be explained by infrequent transcriptional bursting and high mRNA or protein stability. The lineage analysis indicated that in some cases, responding status is maintained for five generations, or 60 h. However, the half-life of TLR2 protein was determined to be ~8 h^35^ (Supplementary Fig. 6e). In addition we sought to mimic a transcriptional burst by pulsing doxycycline for 4 h in our TRE3G::TLR2 cells. After the pulse, the percent of responding cells fell rapidly, and at 72 h, TLR2 expression was undetectable, as in WT cells (Fig. 4e, c). Given that TLR2 is not stable enough to persist for five generations, we propose that epigenetic control of the TLR2 promoter licenses transcription for multiple generations. This additional regulatory layer would enable spatio-temporal control over the fraction of responders by altering the epigenetic switching probabilities at the single-cell level.

### TLR2 promoter methylation correlates with the fraction of responders

**Fig. 5:**
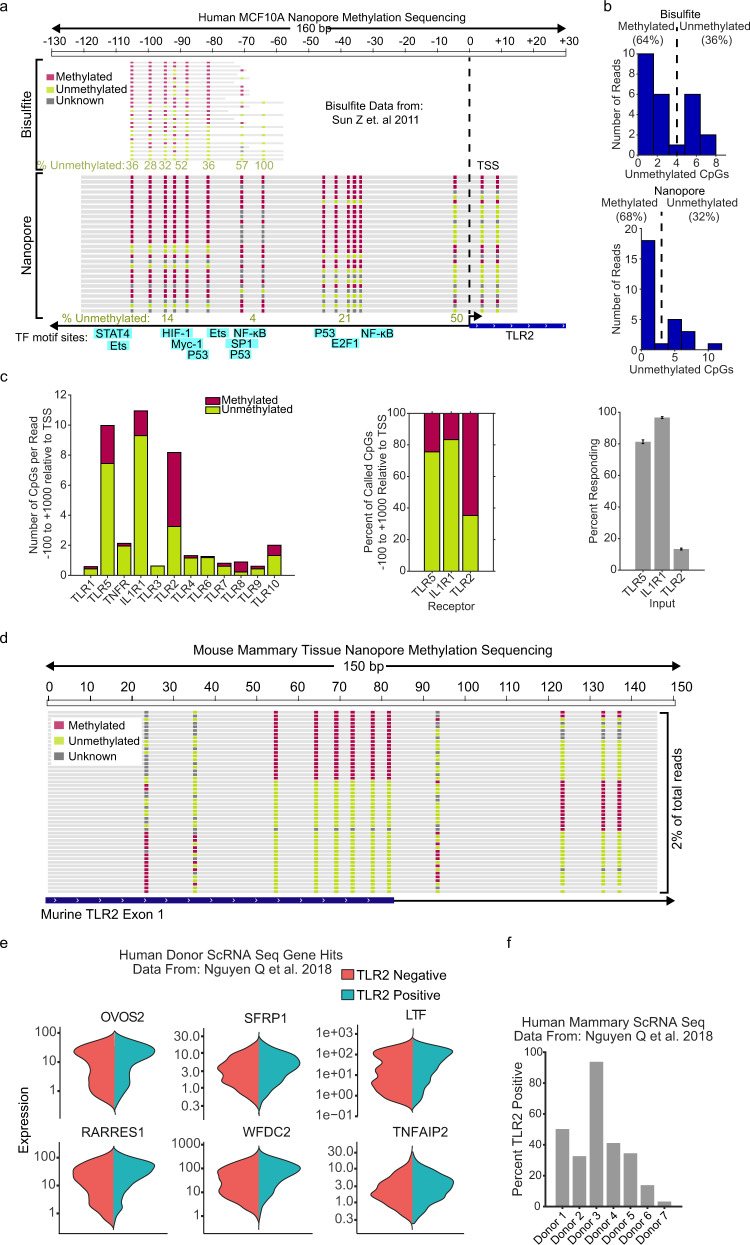
TLR2 promoter methylation is bimodal. **a** Bisulfite^36^ and nanopore sequencing data of 130 bp upstream of the TLR2 transcription start site (TSS) in MCF10A. Locations of transcription factor consensus binding sequence sites are indicated. **b** Histogram quantifications of the number of unmethylated CpGs per read between −110 and −60. **c** Nanopore methylation sequencing^57^ data of 100 bp upstream to 1000 bp downstream of TSS for indicated receptor. Percentage of unmethylated and methylated CpGs as well as percent responding cells as determined by p65 immunofluorescence following TLR5 (Flagellin), IL1R (IL-1B), and TLR2 (Pam) stimulation. **d** TLR2 locus targeted nanopore sequencing of genomic DNA isolated from primary mouse epithelial mammary cells. **e** Differentially expressed genes cosegregating with TLR2 negative and positive cells in publicly available single-cell RNA sequencing data from human breast epithelial tissues^40^. **f** Percent TLR2 positive cells from ScRNA sequencing data collected from breast tissue of seven female donors. Only cells expressing luminal expression markers were considered (see Methods section for details).

Our data suggest that TLR2 expression occurs via epigenetic licensing. Recent studies have used allele-specific methylation to show that DNA methylation occurs in metastable states (e.g. all methylated or all demethylated)^24^. Thus, we wanted to determine if methylation of the TLR2 promoter is bimodal. To address this question, we used raw data from bisulfite^36^ or nanopore sequencing in MCF10A cells and extracted the individual reads aligning to the TLR2 promoter region (from −120 bp to transcription start site, TSS). This region contains multiple CpG sites that have previously been shown to regulate TLR2 expression^37,38^. Our analysis confirmed that single TLR2 reads were methylated en bloc between the −110 and −60 positions (Fig. 5a). In fact, a calculation of the total unmethylated residues in this region showed a clear bimodal distribution with mostly methylated or unmethylated CpGs (Fig. 5b). This data suggests that TLR2 promoter methylation exists in two metastable states. Next we asked whether other cytokine or pattern recognition receptors also showed similar regulatory elements. While most receptors did not contain CpG islands, the TLR5, IL1R1, and TLR2 start sites contain metastable blocks of 10 or more CpG sites. Notably, the percentage of methylation loosely reflected the percentage of NF-κB response to their respective ligands (Fig. 5c and Supplementary Fig. 7).

DNA hypermethylation is known to occur in cultured and transformed cells spontaneously^39^. Thus, we asked whether bimodal states also occur in primary tissues by measuring TLR2 locus methylation in mouse mammary tissue. Interestingly, most of the sequencing reads were unmethylated, agreeing with the fact that in primary organoids the fraction of responders is 50–60%, as opposed to 10–20% in cell lines (Fig. 2c–e). Yet, a fraction of en bloc methylated reads was also present in primary non-cultured tissues (Fig. 2d). This trend indicates that switch-like epigenetic modifications of the TLR2 locus also occur in vivo.

Next, we wanted to investigate if responder cells have other specialized functions within epithelial tissues beyond the detection of TLR2 agonists. We explored single-cell RNA sequencing data from human mammary tissue^40^ to ask if TLR2 expressing cells have other coregulated genes. Results showed that TLR2 positive cells indeed coregulate other genes that are associated with cytokine production and immunity according to standard GO term analysis (Fig. 5e and Supplementary Table 1). Of note, all tissue donors expressed TLR2 in a subset of luminal cells, however the fraction of cells was highly variable between donors (~30%, max 93%, min 3%; Fig. 5f). Next, we decided to further study the regulation of the responder fraction at the tissue level upon genetic or environmental perturbation.

### Environmental and genetic changes increase percent responders

**Fig. 6:**
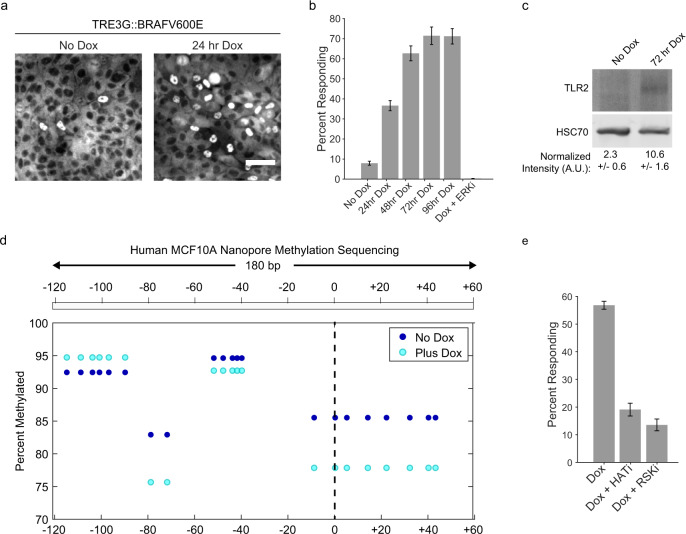
Oncogene-induced epigenetic modifications increase the responder percentage. **a** Clonal MCF10A cell line containing TRE3G::BRAF^V600E^ was immunostained for p65 after 24 h of culture in the presence or absence of Doxycycline (2 µg/ml) after Pam3CSK4 (1 µg/ml) stimulation for 30 min. Representative images are shown. Scale bar, 50 μm. **b** Monolayers treated with Pam3CSK4 (1 µg/ml) only, or increasing duration of dox, or ERKi (5 μM ulixertinib), prior to Pam3CSK4 treatment. Data represents three replicates. **c** TLR2 immunoblotting of BRAF^V600E^ dox inducible cells as in panel **b**. HSC70 is loading control, relative amounts are reported with standard deviation representing two independent experiments. **d** TLR2 locus targeted nanopore methylation sequencing of genomic DNA isolated from human MCF10A cells with (Plus Dox, 96 h) or without (No Dox) BRAFV600E overexpression. **e** TRE3G::BRAF^V600E^ monolayers were cultured with dox or inhibitors (HATi, A-485 (10 µM) and RSKi, BI-D1870 (10 µM)) and percent Pam3CSK4 (1 µg/ml) responders was calculated by NF-κB immunofluorescence. Error bars represent three technical replicates.

One of the hallmarks of oncogenic transformation is widespread epigenetic dysregulation^41^. In particular, the BRAF^V600E^ mutation, present in 50–70% of melanomas, has been shown to broadly alter DNA methylation^42,43^. We asked if the expression of BRAF^V600E^ changes the fraction of responding cells using an inducible promoter. Results showed that the fraction of responders was increased after only 24 h of BRAF^V600E^ expression and reached a new steady state at ~70% of responders in 72 h (Fig. 6a, b). This transition was ERK-activity dependent, as addition of the ERK inhibitor Ulixertinib^44^ completely abolished the change in responder cell fraction (Fig. 6b). Accordingly, TLR2 and TLR1 expression levels were elevated in 72-h lysates, corroborating the link between percent responders and TLR2 (Fig. 6c and S8a). The amplitude of the NF-κB response between expressing and non-expressing BRAF^V600E^ cells is similar, suggesting that while expression is licensed in a higher fraction of cells, the amount of TLR2 function per cell is comparable (Supplementary Fig. 8b). Nanopore sequencing of the TLR2 locus before and after doxycycline addition revealed a reduction in methylation (Fig. 6d). While this result suggests that the increase in responder cells is correlated with a decrease in methylation, the percentage of responders does match the percentage of methylated reads. Thus, we conclude that additional epigenetic modifications or complex temporal dynamics of epigenetic regulatory events are likely to be involved.

Previous studies have suggested that ERK signaling induces epigenetic changes via p90 RSK and the histone acetyltransferase, CBP/p300^45^. To address whether the rapid increase in responders was due to ERK-mediated epigenetic changes, we induced expression in the presence of RSK or histone acetyltransferase CBP/p300 inhibitors. Both inhibitors significantly blocked the non-responder to responder transition (Fig. 6e). Together, our data suggest that genetic perturbations such as oncogenic mutations can alter the epigenetic switching probabilities in individual cells resulting in changes of the fraction of responding cells at the tissue level.

**Fig. 7:**
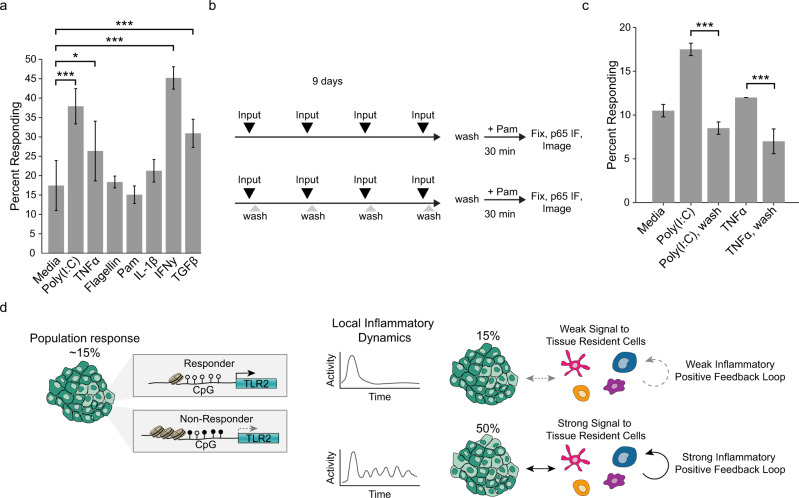
High steady-state NF-ĸB signaling increases the responder percentage. **a** WT monolayers were treated with indicated stimulus four times over a 9-day period. Concentrations of inputs were: Poly(I:C) 1 µg/ml, TNFα 100 ng/ml, Flagellin 1 µg/ml, Pam3CSK4 1 µg/ml, IL-1β 100 ng/ml, IFNγ 5 µ/ml, and TGFβ 5 ng/ml. Error bars represent six technical replicates with *n* > 1000 cells per replicate. Two-sample *t* test *p < 0.05 and ***p < 0.001. **b** Schematic indicating experimental workflow for data in panel **c**. Monolayers were stimulated with inputs every 2 days and washed or not after 30 min. After the last stimulation (day 8), all monolayers were washed for 24 h before determining responder fraction with Pam3CSK4 as in Fig. 2a. **c** Monolayers treated as detailed in panel **b**, input concentrations as in panel **a**. Two biological replicates, five technical replicates for each condition, ***p < 0.001; two-sample *t*-test. **d** Summary of model. Epithelial tissue contains cells that are all-or-nothing responsive or non-responsive to lipopeptide agonists. Response status is defined by TLR2 expression, which is controlled by epigenetic modifications to the TLR2 promoter. At steady state the fraction of responders is low; however, high inflammatory signaling involving innate immune cell types (e.g. dendritic cells, macrophages), leads to changes in the rate of epigenetic switching that increase the fraction of responsive cells in the tissue.

As stated before, we hypothesized that the tissue may be able to adjust its response by altering the fraction of responders (Supplementary Fig. 2b). Because population assays show that TLR2 expression is affected during chronic inflammation^33,34,38^, we speculated that prolonged exposure to cytokines or MAMPs may affect the fraction of responders. To mimic such a scenario, we cultured cells with a panel of innate immune pathway activators for an extended period of time, removed the treatment for 24 h and stimulated with Pam to determine the fraction of responders. Interestingly, we found that MyD88-independent signaling from Poly(I:C), TNFα, IFNγ, and TGFβ significantly increased the upper limit of the responding fraction, while MyD88-dependent Flagellin, Pam, and IL-1β signaling did not (Fig. 7a). The effect of Poly(I:C) and TNFα introduced the intriguing possibility that prolonged NF-κB basal activity contributes to this adjustment of the monolayer while a single pulse of activity created by inputs such as Flagellin, Pam, or IL-1β, does not. We tested this hypothesis by comparing brief stimulation to continuous stimulation (Fig. 7b). We found that only the cells that were stimulated continuously showed a significant increase in the fraction of responder cells (Fig. 7c). These data support the idea that sustained oscillatory NF-κB signaling, generated from receptors that do not signal through MyD88, can increase the TLR2-responsive fraction in the epithelium. Thus, regulation of the TLR2 epigenetic status by certain cytokines spatio-temporally adjusts the upper response limit of the tissue (Fig. 7d).

## Discussion

Single cells are often compared to computers, dutifully interpreting their surroundings to produce reliable responses. While useful for modeling signaling networks, this outlook is not often reflected in biological experiments; even in isogenic cells living in the same environment, noise generated by the transcription and translation machinery impedes production of an output that accurately reflects the strength of the input. Here we propose a new mechanism by which epithelial tissues exert quantitative control over signaling output despite the widespread biological noise: adjustment of the fraction of cells capable of input detection. We show that bacterial lipopeptide signaling through TLR2 is functionally bimodal in cultured human epithelial cell lines and mouse organoids. TLR2 is the most versatile TLR, dimerizing with TLRs 1, 2, 6, and 10, in order to detect a wide variety of bacterial and viral molecular patterns as well as danger signals produced during tissue damage^30^. Therefore, the bimodal regulation of TLR2 expression described here likely has implications beyond the detection of bacterial lipopeptides.

Digital signaling in the NF-κB network has been described as a concentration-dependent switching event where increasing concentrations of stimuli result in activation of increasing fractions of cells^14,28^. We find that in addition to this behavior, the fraction of responsive cells can be limited by an epigenetic licensing event. In fact, live-cell reporters of epigenetically regulated gene expression support a model where CpG island-containing promoters inhabit highly metastable states^24,27^. Accordingly, our analysis of nanopore methylation and bisulfite sequencing data reveal that the TLR2 promoter CpG island is methylated en bloc. Since bimodal signaling events are prevalent in both adaptive and innate immunity^27,28^, we speculate that epigenetic licensing enables tighter coupling of response levels to the larger context of the inflammatory process. Of note, since the majority of inflammatory cytokines are produced by tissue-resident immune cells such as macrophages and dendritic cells, we anticipate that these cells will play a critical role in promoting local epigenetic switching of epithelial cells. Future research will address the role of epigenetic switching in adaptive immunity in vivo.

Long-term incubation with inflammatory signals and BRAF^V600E^ expression shows that the rate of epigenetic switching can be heavily influenced by either environmental or genetic perturbations. Notably, environmental perturbations that trigger switching also elicit high basal NF-κB signaling (Fig. 6). This suggests that during inflammation, sustained NF-κB activity dynamics may trigger positive feedback at the tissue level. In agreement, population-based studies have shown that during chronic inflammatory diseases such as periodontitis, pulmonary tuberculosis, and cystic fibrosis, hypomethylation of the TLR2 promoter leads to enhanced expression of TLR2^33,34,46^. However, understanding this effect at the single-cell level has important conceptual implications that offer new therapeutic strategies to desensitize innate immunity in epithelial tissues and potentially impede the progression of chronic inflammation. Furthermore, considering that both innate and adaptive immunity are subject to epigenetic regulation^27,47^ and are both dominated by digital signaling events, we propose that epigenetic switching could be a general mechanism for limiting tissue- or organism-level inflammation during the immune response.

## Methods

### Cell lines and culture

MCF10A cells were cultured in Dulbecco’s Modified Eagle Medium (DMEM)/F12 (Thermo) supplemented with 5% Horse Serum (HS) (Sigma), 20 ng/ml EGF (Peprotech), 0.5 mg/ml hydrocortisone (Sigma), 100 ng/ml cholera toxin (Sigma), 10 µg/ml insulin (Sigma), 1% Penicillin/Streptomycin (Gibco) at 37 °C, and 5% CO2. HUVEC cells (ATCC CRL-1730) were maintained in F-12K base medium (Thermo) supplemented with 0.1 mg/ml heparin (Sigma), 10% FBS (Sigma), 1% Penicillin/Streptomycin (Gibco), and 0.3 mg/ml Endothelial Cell Growth Supplement (Corning). BJ fibroblasts were cultured in Dulbecco’s Modified Eagle Medium (Thermo) with 10% FBS (Sigma), 1% Glutamax (Thermofisher), and 1% Penicillin/Streptomycin (Gibco). All cell lines were maintained per ATCC instructions.

### Plasmid and cell line generation

Plasmids were produced using Gibson assembly^48^ into a pENTR vector, sequenced, then recombined into a pDEST lentiviral vector using Gateway cloning^49^. Cell lines were established by lentiviral infection. Lentiviruses were generated with lipofectamine transfection of third-generation viral packaging plasmids and lentiviral construct into HEK293FT cells. Cells were incubated in viral supernatant with Polybrene (10 µg/ml; EMD Millipore) before selection with puromycin (Invivogen; 2 µg/ml).

### Live-cell imaging

Cells were imaged with a Nikon Eclipse Ti-E inverted fluorescence microscope with a ×20 objective and Hamamatsu sCMOS camera controlled by Metamorph software. LED excitation light source (SPECTRA X) was used at 430/24 nm (mCerulean3), 470/24 (mClover or GFP), 500/20 (mClover, used when imaged with mCerulean3), 550/15 nm (mRuby2 or dsRed), and 640/30 nm (iRFP670). Exposure times for each light channel were limited to 250 ms and the frequency of imaging acquisition was 5 minutes (unless otherwise stated). Temperature (37 °C), CO2 (5%), and humidity were controlled throughout the experiments.

### Image analysis

Registration and flatfielding of images were performed using custom Matlab software. Object identification and segmentation were achieved using CellProfiler^50^. Nucleus and an expanded cytoplasm ring (cytoring) were segmented for each cell, using iRFP-H2B nuclear marker fluorescence, and quantified in each channel. Custom Matlab and Python software were used to track and curate cells. Thresholding was applied to decrease background fluorescence contribution. For NF-κB nuclear translocation quantification, NF-κB nuclear/cytoplasmic median intensities were used. Cells with initial NF-κB expression outside of a 25–99% range were rejected. Additional image analysis was performed using custom Python and Matlab scripts.

### MAMP screen

Clonal MCF10A cells expressing an H2B-iRFP nuclear marker, p65-mRuby, and containing the reporter (7x NF-κB binding sites driving mVenus-PEST expression) were seeded onto a glass plate coated with fibronectin. Cells were cultured for 48 h to allow for a complete monolayer to form, then full serum media was replaced with imaging media (1% Horse Serum, 1x Penicillin/Streptomycin, HEPES in Phenol-red free DMEM). Cells were allowed to adjust to new media for 1 h. Cells were imaged for 45 min then the indicated input and concentration was added. Imaging continued for 12 h. Innate immune activators were: Human TNFα (Life Technologies, PHC3015), Poly(I:C) (Sigma-Aldrich, P1530), Pam3CSK4 (InvivoGen, tlrl-pms), and IL-1β (R&D Systems, 401-ML-005).

### Immunofluorescence

Cells were treated with indicated input for 30 min and fixed with 4% formaldehyde for 15 min. Cells were washed three times with 1X PBS and then blocked with blocking buffer (1X PBS, 1% BSA (Sigma, A9647), 0.3% Triton™ X-100 (Sigma, T-8787) followed by overnight primary incubation in 1:400 NF-κB p65 monoclonal antibody (Cell Signaling Technologies, D14E12). Secondary antibodies were either Alexa Fluor 405 (Abcam, ab175649) or Cy3 (Jackson ImmunoResearch, 711-165-152).

### Lineage tracing

Lineages were traced from 3 × 3 tiled array movies of MCF10A monoclonal cell line expressing an H2B-iRFP nuclear marker. Monolayers were plated on fibronectin-coated glass plates at low density and imaged every 5 min for 60 h. Divisions were manually tracked and constructed in Matlab using a modified version of software developed by the National Institute of Standards and Technology (NIST)^51^. Cells that died or traveled out of the field of view were marked with gray at the end of the trace.

### Dual mRNA FISH immunofluorescence

TLR2 probes were synthesized in house by a protocol derived from Gaspar et al.^52^. Cy3 fluorescent dye was conjugated to 20 nt DNA oligonucleotides complementary to the human TLR2 transcript using terminal deoxynucleotidyl transferase and dideoxy-UTP (Lumiprobe 15040). MCF10A cells were grown on fibronectin-coated glass coverslips (Corning 354088). smFISH-IF was performed as described in Eliscovich et al.^53^. All solutions were supplemented with 2 mM ribonucleoside vanadyl complex (RVC, NEB S1402S) to prevent RNA degradation. Prior to fixation, cells were washed 3x with 1x PBS (Corning 21-031-CV) + 5 mM MgCl2 (Sigma-Aldrich M2670-500G) (PBSM). Cells were treated and fixed with 4% paraformaldehyde (Electron Microscopy Sciences 50-980-492), washed 3x with PBSM, and permeabilized in 1x PBS (Corning 21-031-CV), 0.1% Triton X-100 (Sigma-Aldrich T8787-100mL), 0.5% BSA (VWRV0332-25G). Cells were again washed 3x with PBSM. The cells were then incubated for 30 minutes at room temperature in a 2x saline-sodium citrate buffer (SSC, Corning 46-020-CM) supplemented with 10% deionized formamide (Sigma-Aldrich F9037-100ML) and 0.5% BSA (VWRV0332-25G). Probes were hybridized at 37 °C for 3 h in hybridization buffer consisting of 10% deionized formamide (Sigma-Aldrich F9037-100ML), 1 mg/ml E. coli tRNA (Sigma-Aldrich 10109541001), 10% dextran sulfate (Sigma-Aldrich D8906-100G), 0.2 mg/ml BSA (Ambicon AM2616), 2X SSC (Corning 46-020-CM), 2 mM RVC (NEB S1402S), 10 U/ml SUPERasIn (Thermo Fisher AM2694), 60 nM TLR2 probe, 60 nM control PolII probe, and 1:400 NF-κB p65 monoclonal antibody (Cell Signaling Technologies, D14E12). After hybridization, cells were washed 4x in 2xSSC (SSC, Corning 46-020-CM) + 10% formamide (Sigma-Aldrich F9037-100ML) and incubated 2x20min at 37 °C in 2xSSC (Corning 46-020-CM) + 10% formamide (Sigma-Aldrich F9037-100ML), supplemented with a 1:1,000 goat anti-rabbit secondary antibody conjugated to Alexa-488 (Life Technologies A11008). After incubation and washing, the coverslips were mounted on microscope slides (Fisher 12-552-3) with Prolong Diamond + DAPI mounting media (Invitrogen P36962) and allowed to cure overnight.

Samples were imaged on a custom wide-field inverted Nikon Ti-2 wide-field microscope equipped with 60×1.4NA oil immersion objective lens (Nikon), Spectra X LED light engine (Lumencor), and Orca 4.0 v2 scMOS camera (Hamamatsu). The x-y pixel size was 107.5 nm. The z-step size was 300 nm.

### Primary mammary and gut organoid cultures

Organoids were isolated from murine mammary glands and mammary tumors using previously described techniques^54^. All mouse procedures were reviewed and approved by the Animal Care and Use Committee (ACUC). Briefly, normal mammary glands were harvested from 8- to 12-week-old mice, mechanically disrupted with a scalpel, and digested on a shaker for 1 h at 37 °C in collagenase solution. The suspension was centrifuged at 400×*g* to remove cellular debris and undigested tissue, and the pellet was treated with DNase. The epithelial organoids were enriched and separated from stromal cells by a series of differential centrifugations, following which organoids of ~100–500 epithelial cells were obtained. To perform 3D culture of primary murine mammary organoids, the isolated organoids were suspended in Matrigel concentration of 1–2 organoids/μl and plated as 100–120 μl suspensions in 24-well glass-bottom plates (655892; Greiner Bio One) over a 37 °C heating block. Matrigel gels were polymerized at 37 °C for 1 h before the addition of organoid medium.

Primary 2D gut monolayers were derived as previously described^31^. Briefly, mouse jejuna were dissected from 4- to 6-month-old mice. Jejuna were sliced lengthwise and incubated 30 min in cold PBS with 100 U/ml penicillin and streptomycin, 1.5 mM DTT, 2 mM EDTA, and 10 μM Y-27632. Jejuna were then transferred to cold PBS with 2 mM EDTA and shaken for 2 min to free crypts. Intestinal tissue was discarded and the crypt suspension was washed three times in DMEM with 10% FBS by centrifugation at 300×*g* for 3 min then the crypts were resuspended in DMEM with 10% FBS and filtered through a 500-μm strainer followed by a 70-μm strainer. The crypts were pelleted, then resuspended in attachment media, which consisted of basal organoid media (advanced DMEM-F-12 medium with 100 U/ml penicillin and streptomycin, 10 mM HEPES buffer, and 1x Glutamax) supplemented with 1 mM *N*-acetyl-cysteine, 1x B27 supplement, 50 ng/ml EGF, 100 nM LDN-193189, 1 μg/ml R-spondin 1, 10 μM CHIR99021, and 10 μM Y-27632 and seeded to optical polymer 96-well plates coated with 0.8 mg/ml Matrigel. Crypts were incubated in attachment media overnight before media was exchanged for organoid culture media (basal organoid media supplemented with 1X B27, 1 mM *N*-acetyl-cysteine, 100 μg/ml primocin, 50 ng/ml EGF, 10% Noggin-conditioned medium, and 20% R-spondin conditioned medium). Enteroids received fresh organoid culture medium every 24 hours and were cultured up to 1 week.

### Western blotting

Monolayers were grown for 2 days unless otherwise specified. Protein was extracted in Lysis Buffer (50 mM Tris pH 7.4, 150 mM NaCl, 1% Triton, 0.5% Na-Deoxycholate, 1% SDS, 2 mM EDTA, 1x fresh Halt Protease and Phosphatase Inhibitor Cocktail (Thermo Fisher Scientific), and 1 mM DTT). Protein was heated in Laemmli sample buffer at 70 °C for 10 min (Bio-Rad), resolved by SDS-PAGE, and transferred to a PVDF membrane (Milipore-Sigma). Blocking and antibody dilutions were done in Odyssey Blocking Buffer (Li-COR) and washes in PBS/Tween-20 (0.1%). Antibodies used were TLR2 (CST 12276) and HSC70 (Santa Cruz 7298). Membranes were read with a Li-COR scanner and quantified using ImageStudioLite software and ImageJ. Relative amounts in the quantification means the median signal with local background subtracted, divided by the loading control for each sample, multiplied by 100 for simplicity.

### Bisulfite, nanopore, and ScRNA sequencing data analysis

Bisulfite sequencing reads were obtained from Sun et al.^36^ (GEO Series GSE27003). Quality control and adapter trimming were performed with TrimGalore1 version 0.6.5 (https://github.com/FelixKrueger/TrimGalore). Alignment to hg38 reference genome and methylation calling were performed using Bismark2^55^ version 0.22.3 with Bowtie23^56^ version 2.4.1. Whole genome nanopore sequencing data and methylation calls (Supplementary Fig. 8b) were kindly provided by Isac Lee and Dr. Timp^57^. Single-cell RNA sequencing expression counts were obtained from Nguyen et al.^40^ (GEO Series GSE113197). For each donor, uniform manifold approximation and projection (UMAP) analysis identified 1-2 KRT18+/KRT14− clusters which were used for analysis of luminal cells. Individual cells were separated based on the presence or absence of TLR2 expression and differential expression analysis was performed on these two groups using Monocle34^58^ version 0.2.1. For differential expression analysis, donors 1–3 were excluded due to low cell numbers and donor 7 was excluded due to a low proportion of TLR2+ cells. Genes which were significantly upregulated (false discovery rate *q* value < 0.05) in the TLR2 + population in at least 2 of the 3 donor samples were included for gene ontology enrichment analysis using the PANTHER^59^ Overrepresentation Test5.

### Targeted nanopore methylation sequencing

Genomic DNA was isolated for nanopore sequencing from mouse mammary tissue and MCF10A. Mouse mammary tissue was enriched for epithelial cells using the aforementioned mammary organoid isolation protocol without the DNase step. DNA was extracted and purified with the Nanobind kit (Circulomics, catalog no. NB-900-001-01) the day before sequencing. DNA was quantified using the Qubit fluorometer (Thermo Fisher Scientific) immediately before performing the assay. Guide RNA were custom designed with IDT software, selecting for the highest on-target score. Guide RNA sequences for mouse and human TLR2 locus are provided in supplementary table 2. Ribonucleoprotein complex was assembled with gRNA, HiFi Cas9 Nuclease V3 (IDT, catalog no. 1081060), and *trans*-activating crRNAs (tracrRNAs; Integrated DNA Technologies (IDT), catalog no. 1072532), and designed to introduce cuts on complementary strands flanking 5–8 kb of the TLR2 promoter region. About 5 μg of input DNA was dephosphorylated with Quick CIP enzyme (New England Biolabs, catalog no. M0508) and cut with pre-assembled Cas9-gRNA complex at 37 °C for 20 min followed by A-tailing with dATP (Zymo, catalog no. D1005) and Taq DNA polymerase (New England Biolabs, catalog no. M0267). Adapters were ligated then libraries were prepped and sequenced on the Oxford Nanopore GridION sequencer, with the Oxford Nanopore Ligation Sequencing Kit (Oxford Nanopore Technologies, catalog no. LSK109). FASTQ reads were generated using GUPPY (v3.2.10 or v4.0.11) base-calling from electrical data. Reads were aligned to the human reference genome (Hg38) or mouse reference genome (GRCm38/mm10) with minimap2 (2.17-r943-dirty)^60^. CpG methylation calling on nanopore data was performed using Nanopolish (v.0.11.1)^61^. High quality methylation calls were filtered for log likelihood ratios >1.5 for methylated and <−1.5 for unmethylated using the nanopore_methylation_utilities tools (https://github.com/timplab/nanopore-methylation-utilities)^57^. The Nanopolish CpG model cannot detect non-CpG methylation or distinguish *k*-mers with a mixture of methylated and unmethylated CpGs, as the training set is limited to completely methylated or unmethylated sequences. In practice, this means that when the CpG sites are within 10 bp of each other, they are assigned the same methylation status to the entire group. Additional details for targeted nanopore sequencing may be found in^62^, and a step-wise protocol is available on protocols.io (https://www.protocols.io/view/cas9-enrichment-for-nanopore-sequencing-68ihhue).

### Reporting summary

Further information on research design is available in the Nature Research Reporting Summary linked to this article.

## Supplementary information

Supplementary Information

Description of Additional Supplementary Files

Supplementary Movie 1

Supplementary Movie 2

Reporting Summary

## Data Availability

Sequencing reads can be found at the BioSample database, BioProject ID PRJNA687543, accession numbers SAMN17150307 and SAMN17150308. Additional imaging data is available upon request.

## Code availability

Example custom scripts for image and sequencing analysis used during this study are available in the Regot lab Github repository (https://github.com/regotlab/DigitalSignaling).
