# Supplementary material for: Epigenetically regulated digital signaling defines epithelial innate immunity at the tissue level
Source: Nat Commun. 2021 Mar 23;12:1836. doi: 10.1038/s41467-021-22070-x (PMC7988009; doi:10.1038/s41467-021-22070-x)
Supplement: Supplementary file 1 — Supplementary Information [file 41467_2021_22070_MOESM1_ESM.pdf]

Epigenetically Regulated Digital Signaling Defines Epithelial Innate Immunity at the Tissue Level

Helen R. Clark, Connor McKenney, Nathan M. Livingston, Ariel Gershman, Seema Sajjan, Isaac S. Chan, Andrew J. Ewald, Winston Timp, Bin Wu, Abhyudai Singh, Sergi Regot

SUPPLEMENTARY INFORMATION

SUPPLEMENTARY FIGURES

Supplementary Figure 1. Reporter Parameters vs. Ratio Parameters Correlation Analysis and Adaptor Knockouts.
Supplementary Figure 2. Monolayers have bimodal response to lipopeptide
Supplementary Figure 3. Clonal expansion and combined inputs.
Supplementary Figure 4. Digital signaling in fibroblast, endothelial, and primary cells.
Supplementary Figure 5. Complete lineage tracing and epigenetic inhibitors Pam response amplitudes.
Supplementary Figure 6. Pam response MyD88 dependence, smFISH supporting data, TLR2 protein turnover quantification.
Supplementary Figure 7. Whole genome nanopore methylation sequencing data of select receptors.
Supplementary Figure 8. TLR2 co-receptor expression and NF-κB amplitude of BRAFV600E monolayers

SUPPLEMENTARY TABLES

Supplementary Table 1. ScRNA GO Analysis of TLR2 Positive Human Mammary Luminal Cells

SUPPLEMENTARY NOTES

Supplementary Model

Supplementary Figure 1

Reporter Parameters vs. Ratio Parameters Correlation Analysis and Adaptor Knockouts

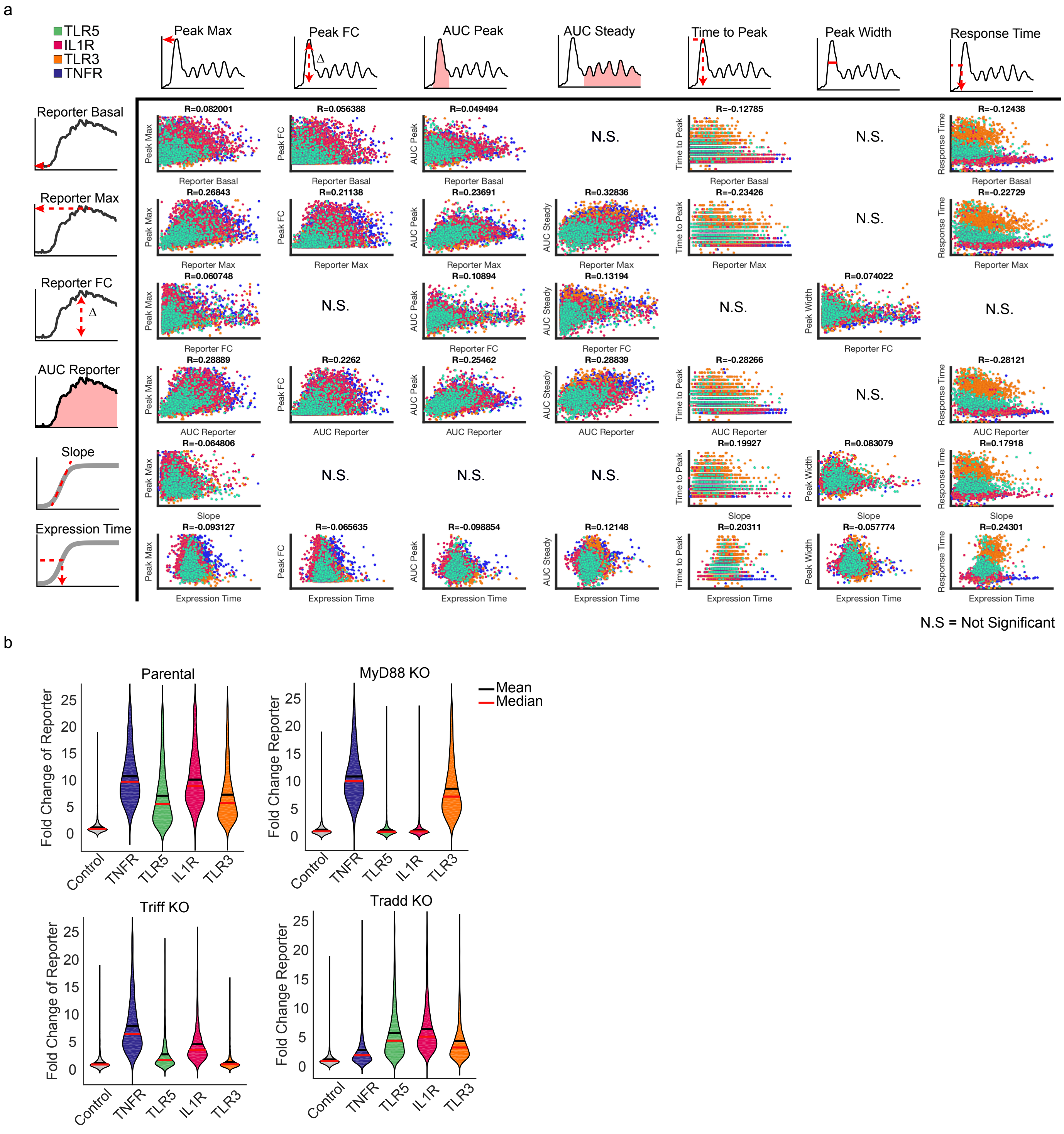

Supplementary Figure 1. Reporter Parameters vs. Ratio Parameters Correlation Analysis and Adaptor Knockouts. (a) Reporter vs. ratio parameters correlation analysis. Scatterplots and correlation coefficients between the parameters that are summarized in the corresponding diagrams. Only those with $p < 0.001$ are shown, plots with p values that were not significant are indicated with N.S. (b) Crispr knockouts of adaptors involved in the NF-κB network (see Fig. 1d). Violin plots show the fold change in reporter from the average of the first five time points to the last five timepoints over a four hour time course after addition of media (control) or inputs that activate the labeled receptors: 100 ng/ml TNFα (TNFR), 1 μg/ml flagellin (TLR5), 100 ng/ml IL-1β (IL1R), 20 μg/ml Poly(I:C) (TLR3).

Supplementary Figure 2

Monolayers have bimodal response to lipopeptide

a

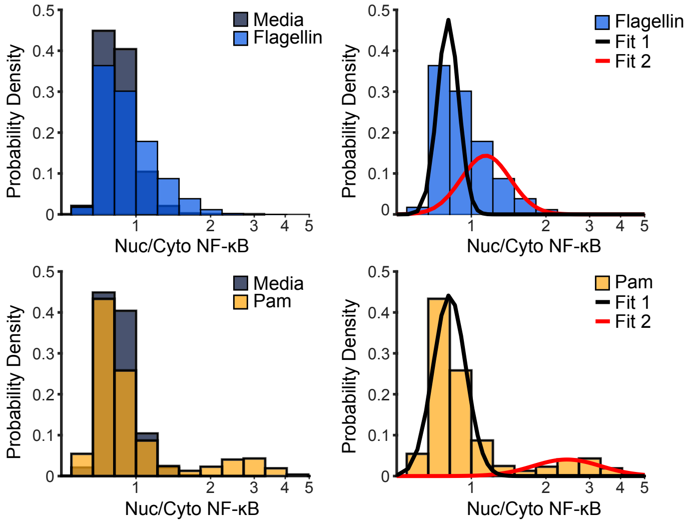

b

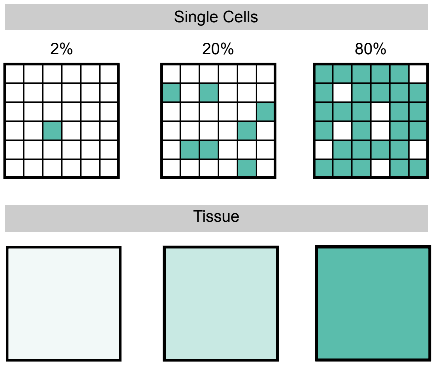

Supplementary Figure 2. Monolayers have bimodal response to lipopeptide (a) Histograms show quantification of the nuclear/cytoplasmic NF-κB response after 1 µg/ml flagellin or 1 µg/ml Pam3CSK4 and were fit to a two-gaussian model. The entropy for two-gaussian fit was 0.42 for flagellin and Pam3CSK4 was 0.04. (b) Schematic showing rationale behind controlling tissue level response by licensing response in single cells. In theory this type of regulatory mechanism would mitigate biological noise from single cells.

Supplementary Figure 3

Clonal expansion and combined inputs

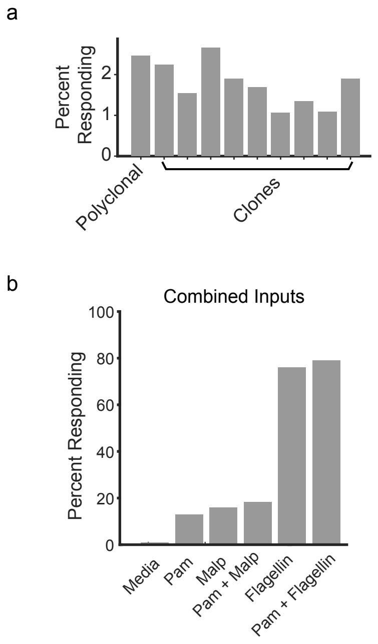

Supplementary Figure 3. Clonal expansion and combined inputs. (a) Percent responders in clones derived from the NF-κB reporter MCF10A cell line. To determine steady state response, clones were cultured for 30 days before percent responders was determined by treatment with 30 minutes 1 μg/ml Pam3CSK4 and NF-κB immunofluorescence. (b) Monolayers were either treated with single inputs or inputs that were combined prior to treatment (1 μg/ml Pam3CSK4, 1 μg/ml MALP, 1 μg/ml flagellin), and were immunostained for NF-κB response.

Supplementary Figure 4

Digital signaling in various cell types

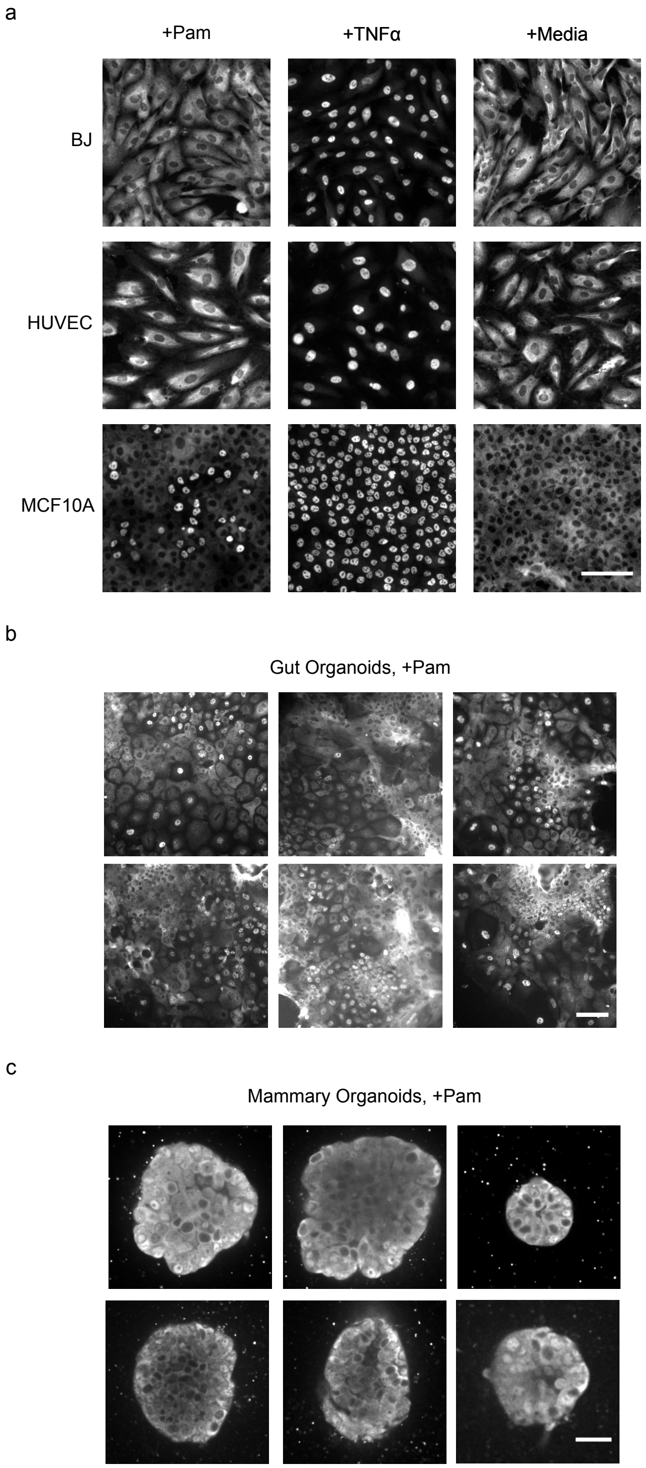

Supplementary Figure 4. Digital signaling in fibroblast, endothelial, and primary cells. (a) BJ, HUVECs and MCF10A were treated with 1 µg/ml Pam3CSK4, 100 ng/ml TNFα, or media, fixed and stained for NF-κB to determine nuclear translocation. TNFα was added 20 minutes prior to fixation while Pam3CSK4 was 30. Scale bar, 100 µm. (b) Gut monolayers were isolated from mouse and grown in 2D on matrigel prior to treatment with 10 µg/ml Pam3CSK4 and immunofluorescent staining for NF-κB translocation. Scale bar, 100 µm. (c) Mammary organoids isolated from mice were embedded in matrigel and grown in 3D prior to treatment with 10 µg/ml Pam3CSK4 and immunofluorescent staining for NF-κB translocation. Scale bar, 10 µm.

Supplementary Figure 5

Complete lineage tracing and epigenetic inhibitors pam response amplitudes

a

b

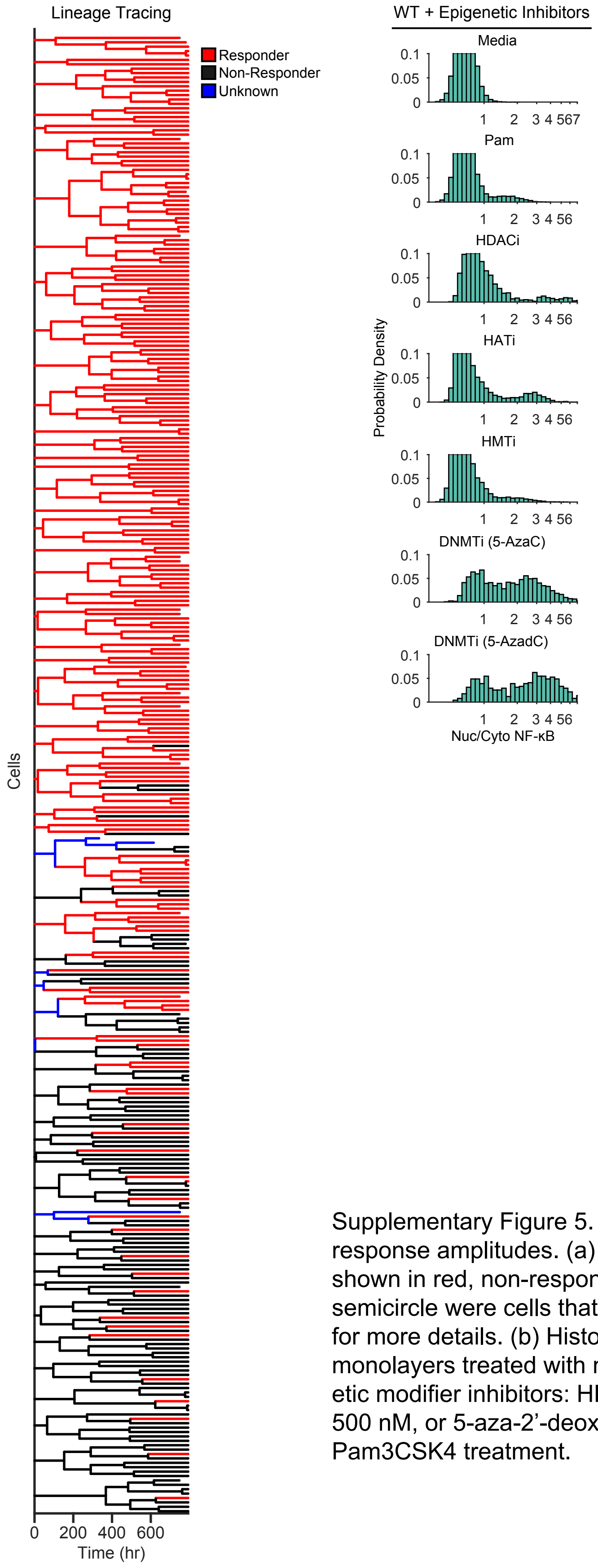

Supplementary Figure 5. Complete lineage tracing and epigenetic inhibitors pam response amplitudes. (a) Complete lineage tracing experiment. Responders are shown in red, non-responders in black, unknown in blue. Traces that end in a grey semicircle were cells that either died or moved out of the field of view. See methods for more details. (b) Histograms of nuclear/cytoplasmic NF-κB response in WT monolayers treated with media, Pam3CSK4 alone, or or were cultured with epigenetic modifier inhibitors: HDACi (SAHA 800 nM), DNMT inhibitors (5-AzacytidineC, 500 nM, or 5-aza-2’-deoxycytidine, 1 μM) HATi (A-485, 10 μM), for a week prior to Pam3CSK4 treatment.

Supplementary Figure 6

Pam response MyD88 dependence, smFISH supporting data, TLR2 protein turnover quantification

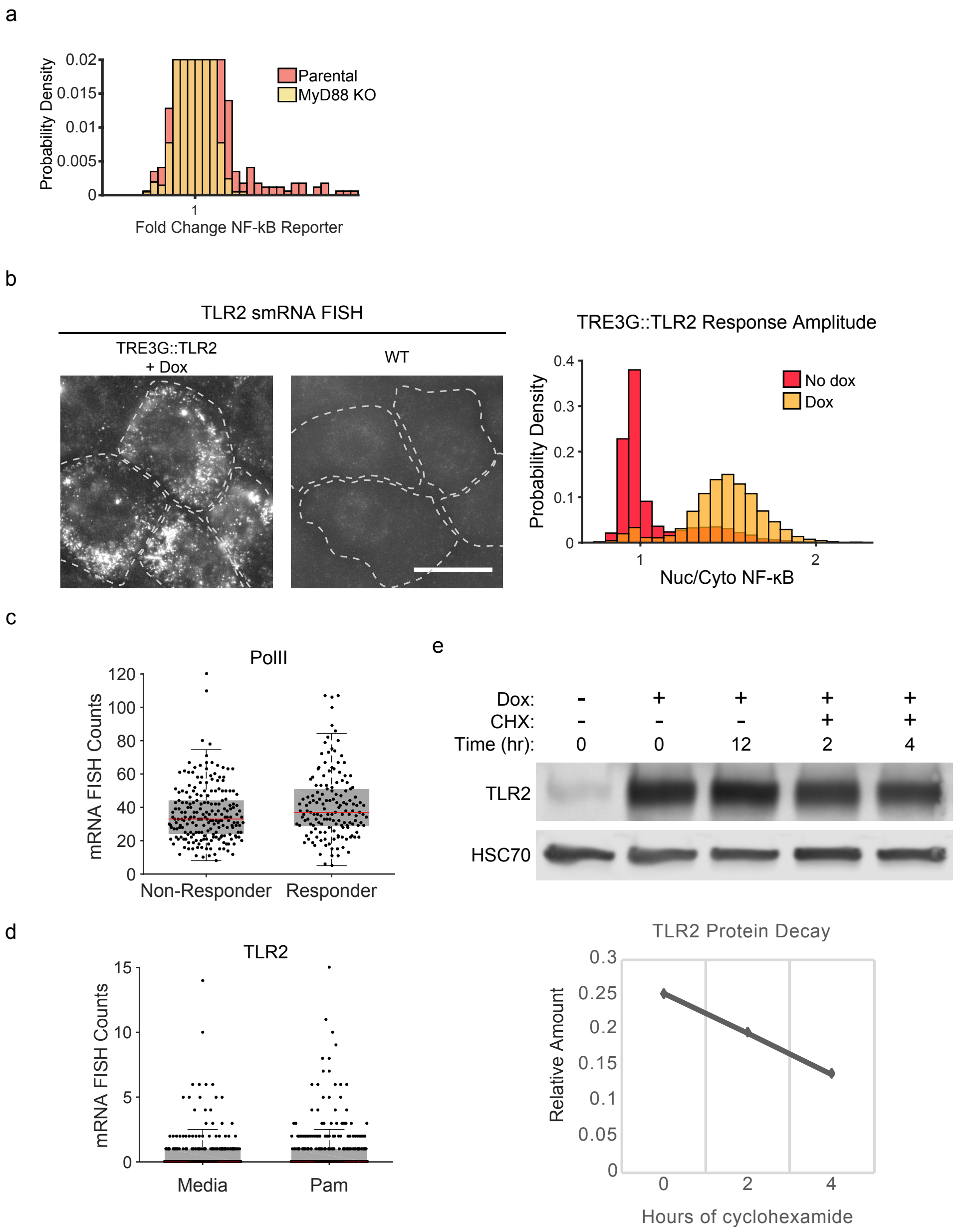

Supplementary Figure 6. Pam response MyD88 dependence, smFISH supporting data, TLR2 protein turnover quantification. (a) NF-κB reporter cell line MCF10A monolayers and the same cell line with MyD88 Crispr deletion were treated with 1 μg/mlPam3CSK4. The fold change of the reporter from the average of the first five time points to the last five timepoints over a four hour time course was determined for each cell. (b) smFISH for TLR2 in 24 hour dox (2 μg/ml) treated tet inducible TLR2 cells and WT cells. Histogram shows amplitude of Pam3CSK4 response in tet inducible TLR2 cells after 24 hours of dox (2 μg/ml) (dox n= 6419 cells no dox n= 7058 cells) . (c) Quantification of mRNA FISH puncta for PolII in Non-Responder and Responder cells as determined by NF-κB immunofluorescence. (d) Quantification of TLR2 mRNA FISH puncta in Pam3CSK4 treated and untreated cells. (e) Immunoblot for TLR2 after overnight dox (2 μg/ml) treatment in tet inducible TLR2 cell line. Before lysate collection, monolayers were treated with or without 20 μg/ml cyclohexamide (CHX) for the indicated number of hours. Quantification of protein relative to HSC70 loading control is shown below.

Supplementary Figure 7

Whole genome nanopore methylation sequencing

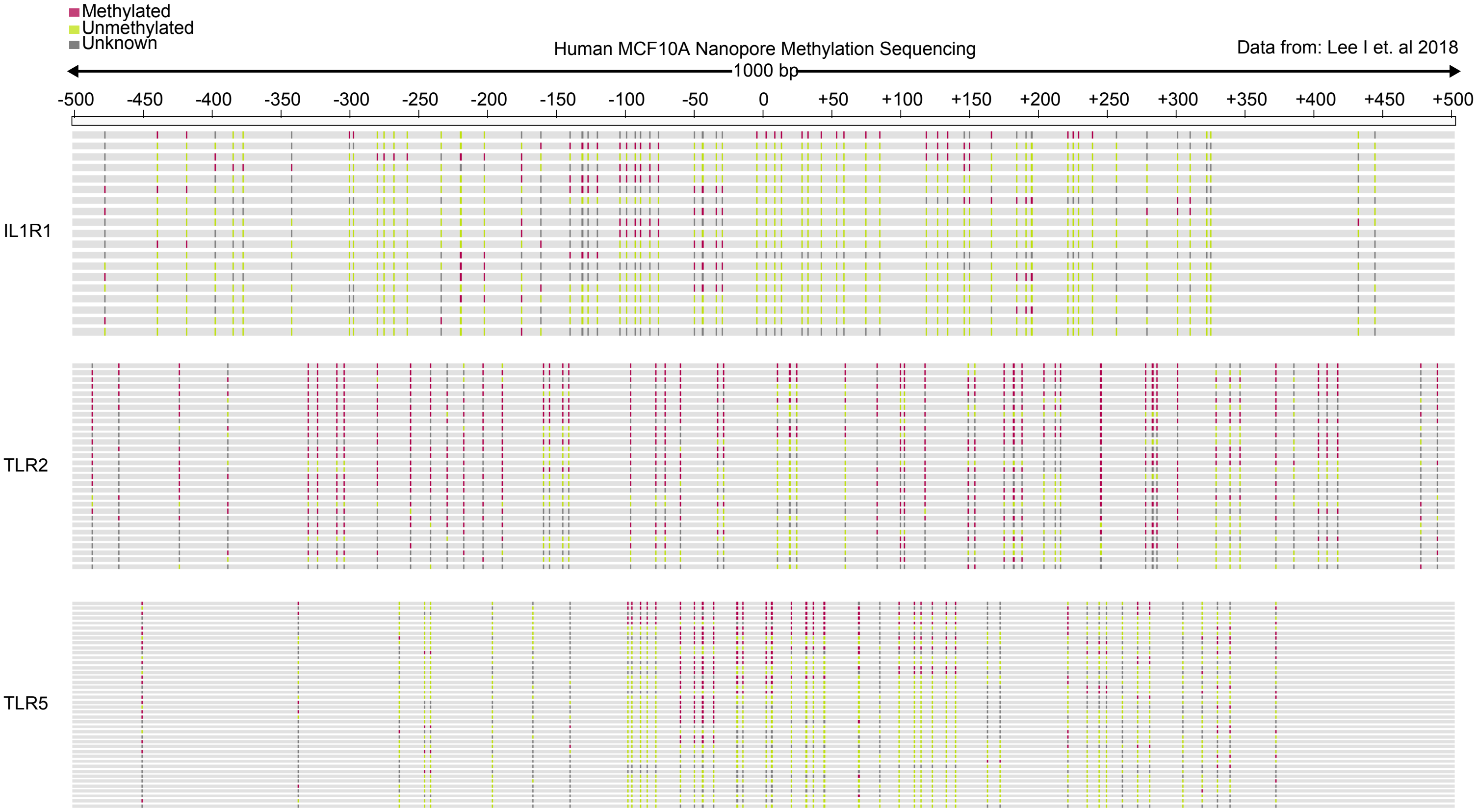

Supplementary Figure 7. Whole genome nanopore methylation sequencing data of select receptors, DNA isolated from MCF10A cells. Data was obtained from Lee I et. al 2018.

Supplementary Figure 8

TLR2 co-receptor expressions NF-κB amplitude of BRAFV600E monolayers

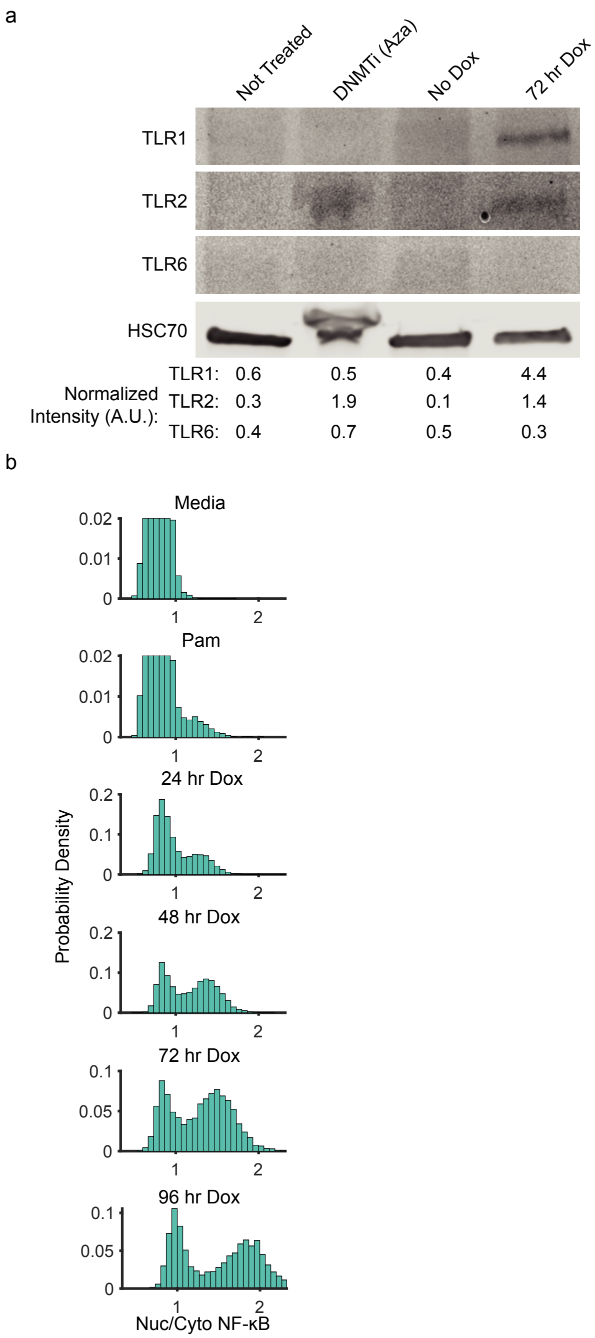

Supplementary Figure 8. TLR2 co-receptor expression and NF-κB amplitude of BRAFV600E monolayers (a) Immunoblot for TLR1, TLR2, TLR6 after DNMTi (5-AzacytidineC, 500 nM) or 72hr dox (2 μg/ml) treatment. Quantification of protein is normalized to HSC70 loading control. (b) Tet inducible BRAFV600E monolayers were treated with media, 1 μg/ml Pam3CSK4 alone, or with increasing durations of doxycycline (2 μg/ml). Histograms show NF-κB nuclear/cytoplasmic amplitude, n > 5000 cells per condition.

Supplementary Table 1

ScRNA GO Analysis of TLR2 Positive Human Mammary Luminal Cells

Analysis Type:	PANTHER Overrepresentation Test (Released 20200407)
Annotation Version and Release Date:	GO Ontology database DOI: 10.5281/zenodo.3727280 Released 2020-03-23
Analyzed List:	TLR2 (Homo sapiens)
Reference List:	Homo sapiens (all genes in database)
Test Type:	FISHER
Correction:	FDR

GO database hit (Biological Process / Cellular Component / Reactome Pathways)	REFLIST (20851)	34	Expected	Over/under	Fold Enrichment	Raw P-Value	FDR
iron import into cell (GO:0033212)	6	2	0.01	+	> 100	7.16E-05	0.0254
cobalt ion transport (GO:0006824)	7	2	0.01	+	> 100	9.19E-05	0.0312
response to mycotoxin (GO:0010046)	7	2	0.01	+	> 100	9.19E-05	0.0306
sequestering of iron ion (GO:0097577)	7	2	0.01	+	> 100	9.19E-05	0.03
response to folic acid (GO:0051593)	10	2	0.02	+	> 100	0.000168	0.0497
negative regulation of osteoclast differentiation (GO:0045671)	29	3	0.05	+	63.44	1.89E-05	0.00863
iron ion transport (GO:0006826)	74	5	0.12	+	41.44	1.74E-07	0.000252
cellular iron ion homeostasis (GO:0006879)	66	4	0.11	+	37.17	4.97E-06	0.00265
iron ion homeostasis (GO:0055072)	83	5	0.14	+	36.94	2.99E-07	0.000341
negative regulation of myeloid leukocyte differentiation (GO:0002	51	3	0.08	+	36.07	9.24E-05	0.0295
transition metal ion transport (GO:0000041)	124	6	0.2	+	29.67	6.04E-08	0.000964
negative regulation of leukocyte differentiation (GO:1902106)	103	4	0.17	+	23.82	2.68E-05	0.0113
transition metal ion homeostasis (GO:0055076)	132	5	0.22	+	23.23	2.7E-06	0.00187
cellular transition metal ion homeostasis (GO:0046916)	109	4	0.18	+	22.51	3.32E-05	0.0136
regulation of myeloid leukocyte differentiation (GO:0002761)	122	4	0.2	+	20.11	5.09E-05	0.0193
negative regulation of hemopoiesis (GO:1903707)	143	4	0.23	+	17.15	9.27E-05	0.029
negative regulation of endopeptidase activity (GO:0010951)	252	6	0.41	+	14.6	3.37E-06	0.00207
negative regulation of peptidase activity (GO:0010466)	262	6	0.43	+	14.04	4.2E-06	0.00239
neutrophil degranulation (GO:0043312)	482	9	0.79	+	11.45	6.34E-08	0.000506
neutrophil activation involved in immune response (GO:0002283)	486	9	0.79	+	11.36	6.8E-08	0.000362
neutrophil mediated immunity (GO:0002446)	493	9	0.8	+	11.2	7.66E-08	0.000306
neutrophil activation (GO:0042119)	495	9	0.81	+	11.15	7.92E-08	0.000253
granulocyte activation (GO:0036230)	500	9	0.82	+	11.04	8.62E-08	0.000229
leukocyte degranulation (GO:0043299)	504	9	0.82	+	10.95	9.21E-08	0.00021
myeloid leukocyte mediated immunity (GO:0002444)	514	9	0.84	+	10.74	1.09E-07	0.000217
myeloid cell activation involved in immune response (GO:000227	519	9	0.85	+	10.63	1.18E-07	0.000209
negative regulation of proteolysis (GO:0045861)	360	6	0.59	+	10.22	2.47E-05	0.0107
myeloid leukocyte activation (GO:0002274)	583	9	0.95	+	9.47	3.1E-07	0.000329
leukocyte activation involved in immune response (GO:0002366)	619	9	1.01	+	8.92	5.08E-07	0.000477
cell activation involved in immune response (GO:0002263)	623	9	1.02	+	8.86	5.36E-07	0.000475
regulation of endopeptidase activity (GO:0052548)	425	6	0.69	+	8.66	6.15E-05	0.0223
regulation of peptidase activity (GO:0052547)	455	6	0.74	+	8.09	8.91E-05	0.0309
leukocyte mediated immunity (GO:0002443)	762	10	1.24	+	8.05	2.63E-07	0.000323
negative regulation of hydrolase activity (GO:0051346)	461	6	0.75	+	7.98	9.57E-05	0.0294
regulated exocytosis (GO:0045055)	696	9	1.13	+	7.93	1.33E-06	0.000965
exocytosis (GO:0006887)	788	10	1.28	+	7.78	3.57E-07	0.000356
leukocyte activation (GO:0045321)	921	11	1.5	+	7.32	1.47E-07	0.000234
cell activation (GO:0001775)	1068	11	1.74	+	6.32	6.34E-07	0.000533
immune effector process (GO:0002252)	1083	11	1.77	+	6.23	7.27E-07	0.000581
secretion by cell (GO:0032940)	1002	10	1.63	+	6.12	3.09E-06	0.00205
cellular response to cytokine stimulus (GO:0071345)	1031	10	1.68	+	5.95	3.97E-06	0.00235
export from cell (GO:0140352)	1049	10	1.71	+	5.85	4.63E-06	0.00255
secretion (GO:0046903)	1119	10	1.82	+	5.48	8.16E-06	0.00407
response to cytokine (GO:0034097)	1121	10	1.83	+	5.47	8.29E-06	0.00401
ion transport (GO:0006811)	1342	10	2.19	+	4.57	3.91E-05	0.0152
establishment of localization in cell (GO:0051649)	2387	16	3.89	+	4.11	2.58E-07	0.000343
immune response (GO:0006955)	1872	12	3.05	+	3.93	2.33E-05	0.0103
vesicle-mediated transport (GO:0016192)	1944	12	3.17	+	3.79	3.39E-05	0.0135
cellular localization (GO:0051641)	2999	17	4.89	+	3.48	9.81E-07	0.000746
cellular response to organic substance (GO:0071310)	2400	13	3.91	+	3.32	5.59E-05	0.0208
immune system process (GO:0002376)	2778	15	4.53	+	3.31	1.13E-05	0.00532
transport (GO:0006810)	4550	20	7.42	+	2.7	3.33E-06	0.00212
establishment of localization (GO:0051234)	4680	20	7.63	+	2.62	5.26E-06	0.00271
localization (GO:0051179)	5802	20	9.46	+	2.11	0.000166	0.05
MHC class II protein complex (GO:0042613)	19	2	0.03	+	64.55	0.00053	0.0425
tertiary granule lumen (GO:1904724)	55	4	0.09	+	44.6	2.5E-06	0.000502
specific granule lumen (GO:0035580)	62	4	0.1	+	39.57	3.93E-06	0.000717
ER to Golgi transport vesicle membrane (GO:0012507)	61	3	0.1	+	30.16	0.000153	0.0162
ficolin-1-rich granule (GO:0101002)	124	4	0.2	+	19.78	5.41E-05	0.00776
ficolin-1-rich granule lumen (GO:1904813)	124	4	0.2	+	19.78	5.41E-05	0.00724
COPII-coated ER to Golgi transport vesicle (GO:0030134)	94	3	0.15	+	19.57	0.000524	0.0438
secretory granule lumen (GO:0034774)	320	8	0.52	+	15.33	4.32E-08	1.24E-05
specific granule (GO:0042581)	160	4	0.26	+	15.33	0.000141	0.0158
cytoplasmic vesicle lumen (GO:0060205)	324	8	0.53	+	15.14	4.75E-08	1.19E-05
vesicle lumen (GO:0031983)	326	8	0.53	+	15.05	4.97E-08	1.11E-05
tertiary granule (GO:0070820)	164	4	0.27	+	14.96	0.000155	0.0156
secretory granule (GO:0030141)	849	9	1.38	+	6.5	6.64E-06	0.00111
extracellular exosome (GO:0070062)	2098	22	3.42	+	6.43	2.06E-14	2.06E-11
extracellular vesicle (GO:1903561)	2119	22	3.46	+	6.37	2.52E-14	1.69E-11
extracellular organelle (GO:0043230)	2124	22	3.46	+	6.35	2.65E-14	1.33E-11
lytic vacuole (GO:0000323)	708	7	1.15	+	6.06	0.000128	0.0161
lysosome (GO:0005764)	708	7	1.15	+	6.06	0.000128	0.0152
secretory vesicle (GO:0099503)	1017	9	1.66	+	5.43	2.77E-05	0.00428
vacuole (GO:0005773)	809	7	1.32	+	5.31	0.000289	0.0252
extracellular space (GO:0005615)	3349	26	5.46	+	4.76	1.15E-14	2.31E-11
extracellular region (GO:0005576)	4381	27	7.14	+	3.78	5.96E-13	2.39E-10
vesicle (GO:0031982)	3893	23	6.35	+	3.62	5.94E-10	1.99E-07
cytoplasmic vesicle (GO:0031410)	2397	12	3.91	+	3.07	0.000257	0.0246
intracellular vesicle (GO:0097708)	2400	12	3.91	+	3.07	0.00026	0.0238
Metal sequestration by antimicrobial proteins (R-HSA-6799990)	6	2	0.01	+	> 100	7.16E-05	0.0234
Peptide chain elongation (R-HSA-156902)	89	4	0.15	+	27.56	1.54E-05	0.00881
Eukaryotic Translation Elongation (R-HSA-156842)	94	4	0.15	+	26.1	0.000019	0.00867
Neutrophil degranulation (R-HSA-6798695)	478	9	0.78	+	11.55	5.91E-08	0.000135
Infectious disease (R-HSA-5663205)	465	6	0.76	+	7.91	0.0001	0.0286
Innate Immune System (R-HSA-168249)	1105	10	1.8	+	5.55	7.31E-06	0.00556
Disease (R-HSA-1643685)	1127	9	1.84	+	4.9	6.15E-05	0.0234
Immune System (R-HSA-168256)	2159	14	3.52	+	3.98	3.1E-06	0.00353

SUPPLEMENTARY NOTES

We consider a model where single cells reversibly switch between two states – responder and non-responder. Starting from a single cell, the lineage expansion is assumed to occur exponentially with rate λ , i.e., the average number of cells in a lineage at time *t* is $e^{\lambda t}$. During the lineage expansion, the mother cell state is inherited by both daughters. Let the switching rate from non-responder to responder be k_{on}, and k_{off} be the switching rate from responder to non-responder. We define dimensionless switching rates

$$\hat{k}_{on} = \frac{k_{on}}{\lambda}, \quad \hat{k}_{off} = \frac{k_{off}}{\lambda}, \tag{1}$$

and in the limit $\hat{k}_{on}$, $\hat{k}_{off} \ll 1$, these dimensionless rates correspond to switching probabilities per generation. At equilibrium, the fraction of responders is given by

$$f = \frac{\hat{k}_{on}}{\hat{k}_{on}+\hat{k}_{off}} \tag{2}$$

and is assumed to be *f = 15%* . Starting from a single responder cell at time *t = 0*, the fraction of responders during the lineage expansion varies as

$$p_r = f + (1-f)e^{-(\hat{k}_{on}+\hat{k}_{off})T} \tag{3}$$

where $T = \frac{t}{\lambda}$ denotes the time in number of generations. Similarly, starting from a non-responder, the fraction of responders at time *T* is

$$p_{nr} = f(1 - e^{-(\hat{k}_{on}+\hat{k}_{off})T}). \tag{4}$$

Having defined the modeling framework, we next consider lineage data across *M* independent lineages, each starting from a single cell that could either be a responder with probability *f*, or a non-responder with probability *1-f*. For each lineage $i \in \{1,2,\dots,M\}$, the number of cells N_i, and the number of responders N_i^r are measured at the end of the experiment. Given the probability of being a responder as derived in (3)-(4), the likelihood of observing N_i^r responders out of N_i cells follows the binomial distribution

$$P(N_i^r|N_i) = f\, p_r^{N_i^r}(1-p_r)^{N_i-N_i^r} + (1-f)\, p_{nr}^{N_i^r}(1-p_{nr})^{N_i-N_i^r} \tag{5}$$

where the first (second) term corresponds to the initial cell being a responder (non-responder). We further condition this probability on having at least one responder

$$P(N_i^r|N_i, N_i^r \geq 1) = \frac{f\, p_r^{N_i^r}(1-p_r)^{N_i-N_i^r} + (1-f)\, p_{nr}^{N_i^r}(1-p_{nr})^{N_i-N_i^r}}{1-f\,(1-p_r)^{N_i}-(1-f)\,(1-p_{nr})^{N_i}}. \tag{6}$$

This leads to the following likelihood function across all lineages

$$\prod_{i=1}^{M} P(N_i^r|N_i, N_i^r \geq 1)$$

which is maximized to obtain estimates of $\hat{k}_{on}$, $\hat{k}_{off}$ for a given fraction of responders *f* as per (2). To obtain 95% confidence intervals, we use bootstrapping were randomized lineage data is generated from the original data, and rates are estimated using maximum likelihood.
