# Supplementary material for: Epigenetically regulated digital signaling defines epithelial innate immunity at the tissue level
Source: Nat Commun. 2021 Mar 23;12:1836. doi: 10.1038/s41467-021-22070-x (PMC7988009; doi:10.1038/s41467-021-22070-x)
Supplement: Supplementary file 2 — Description of Additional Supplementary Files [file 41467_2021_22070_MOESM2_ESM.pdf]

Description of Additional Supplementary Files

Supplementary Movie 1: NF-κB translocation and gene expression reporter. MCF10A reporter cell line was imaged for 50 minutes to establish a signaling baseline, then was treated with 100 ng/ml TNFα and imaged. NF-κB translocation reporter (p65-mRuby) is shown in grey, NF-κB gene expression reporter (Venus-PEST) in green.

Supplementary Movie 2: NF-κB gene expression reporter movies in tandem for all MAMP screen inputs as in Figure 1 e and g. MCF10A reporter cell line was treated with flagellin (100 μg/ml), IL-1β (100 ng/ml), poly(I:C) (20 μg/ml), TNFα (100 ng/ml), or Pam3CSK4 (1 μg/ml) at time zero and imaged.
