# Supplementary material for: Epigenetically regulated digital signaling defines epithelial innate immunity at the tissue level
Source: Nat Commun. 2021 Mar 23;12:1836. doi: 10.1038/s41467-021-22070-x (PMC7988009; doi:10.1038/s41467-021-22070-x)
Supplement: Supplementary file 5 — Reporting Summary [file 41467_2021_22070_MOESM5_ESM.pdf]

nature research

Corresponding author(s): Sergi Regot, PhD

Last updated by author(s): Jan 3, 2021

Reporting Summary

Nature Research wishes to improve the reproducibility of the work that we publish. This form provides structure for consistency and transparency in reporting. For further information on Nature Research policies, see our Editorial Policies and the Editorial Policy Checklist.

Statistics

For all statistical analyses, confirm that the following items are present in the figure legend, table legend, main text, or Methods section.

n/a	Confirmed	
☐	☒	The exact sample size (n) for each experimental group/condition, given as a discrete number and unit of measurement
☐	☒	A statement on whether measurements were taken from distinct samples or whether the same sample was measured repeatedly
☐	☒	The statistical test(s) used AND whether they are one- or two-sided<br>*Only common tests should be described solely by name; describe more complex techniques in the Methods section.*
☒	☐	A description of all covariates tested
☒	☐	A description of any assumptions or corrections, such as tests of normality and adjustment for multiple comparisons
☐	☒	A full description of the statistical parameters including central tendency (e.g. means) or other basic estimates (e.g. regression coefficient) AND variation (e.g. standard deviation) or associated estimates of uncertainty (e.g. confidence intervals)
☒	☐	For null hypothesis testing, the test statistic (e.g. F, t, r) with confidence intervals, effect sizes, degrees of freedom and P value noted<br>*Give P values as exact values whenever suitable.*
☒	☐	For Bayesian analysis, information on the choice of priors and Markov chain Monte Carlo settings
☒	☐	For hierarchical and complex designs, identification of the appropriate level for tests and full reporting of outcomes
☒	☐	Estimates of effect sizes (e.g. Cohen's d, Pearson's r), indicating how they were calculated

Our web collection on statistics for biologists contains articles on many of the points above.

Software and code

Policy information about availability of computer code

Data collection	Data was collected using Metamorph and analyzed with CellProfiler version 3.1.9 and above
Data analysis	Custom code was written in MATLAB version 2016 and 2020, Python 3.6. Nanopore sequencing was done using GUPPY v.3.0.3 for base-calling and Nanopolish v.0.11.1 for CpG methylation calling.

For manuscripts utilizing custom algorithms or software that are central to the research but not yet described in published literature, software must be made available to editors and reviewers. We strongly encourage code deposition in a community repository (e.g. GitHub). See the Nature Research guidelines for submitting code & software for further information.

Data

Policy information about availability of data

All manuscripts must include a data availability statement. This statement should provide the following information, where applicable:

- Accession codes, unique identifiers, or web links for publicly available datasets
- A list of figures that have associated raw data
- A description of any restrictions on data availability

Sequencing reads can be found at the BioSample database, BioProject ID PRJNA687543, accession numbers SAMN17150307 (https://www.ncbi.nlm.nih.gov/biosample/17150307) and SAMN17150308 (https://www.ncbi.nlm.nih.gov/biosample/17150308). Additional imaging data is available upon request.

Field-specific reporting

Please select the one below that is the best fit for your research. If you are not sure, read the appropriate sections before making your selection.

- [x] Life sciences
- [ ] Behavioural & social sciences
- [ ] Ecological, evolutionary & environmental sciences

For a reference copy of the document with all sections, see nature.com/documents/nr-reporting-summary-flat.pdf

Life sciences study design

All studies must disclose on these points even when the disclosure is negative.

Sample size	Sample size was reported as the number of single cells quantified, as well as the number of experimental and technical replicates.
Data exclusions	No data was excluded
Replication	Key results were replicated more than three times and supplemental results were replicated at least twice except for nanopore sequencing. All attempts at replication were successful.
Randomization	Our study did not include experiments that require randomization.
Blinding	Our study did not include results that could be affected by the knowledge of participants, therefore it did not require blinding.

Reporting for specific materials, systems and methods

We require information from authors about some types of materials, experimental systems and methods used in many studies. Here, indicate whether each material, system or method listed is relevant to your study. If you are not sure if a list item applies to your research, read the appropriate section before selecting a response.

Materials & experimental systems

n/a	Involved in the study	
[ ]	[x]	Antibodies
[ ]	[x]	Eukaryotic cell lines
[x]	[ ]	Palaeontology and archaeology
[ ]	[x]	Animals and other organisms
[x]	[ ]	Human research participants
[x]	[ ]	Clinical data
[x]	[ ]	Dual use research of concern

Methods

n/a	Involved in the study	
[x]	[ ]	ChIP-seq
[x]	[ ]	Flow cytometry
[x]	[ ]	MRI-based neuroimaging

Antibodies

Antibodies used	TLR2 (CST 12276) and HSC70 (Santa Cruz 7298), NF-κB (Cell Signaling Technologies, D14E12), Alexa Fluor 405 (Abcam, ab175649), Cy3 (Jackson ImmunoResearch, 711-165-152)
Validation	All antibodies were validated by the manufacturer.

Eukaryotic cell lines

Policy information about cell lines

Cell line source(s)	MCF10A, RWPE1, BJ5T and HUVECs were obtained from ATCC
Authentication	Cells were successfully validated using STR profiling
Mycoplasma contamination	Cells were regularly tested for mycoplasma contamination. All tests were negative.
Commonly misidentified lines (See ICLAC register)	*Name any commonly misidentified cell lines used in the study and provide a rationale for their use.*

Animals and other organisms

Policy information about studies involving animals; ARRIVE guidelines recommended for reporting animal research

Laboratory animals	Mice were females of CD1 or FVB (mammary organoids) background, between 3 and 8 months of age.

Wild animals	*Provide details on animals observed in or captured in the field; report species, sex and age where possible. Describe how animals were caught and transported and what happened to captive animals after the study (if killed, explain why and describe method; if released, say where and when) OR state that the study did not involve wild animals.*
Field-collected samples	*For laboratory work with field-collected samples, describe all relevant parameters such as housing, maintenance, temperature, photoperiod and end-of-experiment protocol OR state that the study did not involve samples collected from the field.*
Ethics oversight	All mouse protocols were approved by the Animal Care and Use Committe at Johns Hopkins University

Note that full information on the approval of the study protocol must also be provided in the manuscript.
